# Fragility fracture, atypical femoral fracture, and osteonecrosis of jaw after bisphosphonate prescription for three and five years, based on primary and secondary care data in England: nested case-control and cohort studies

**DOI:** 10.1136/bmjmed-2025-002085

**Published:** 2026-04-22

**Authors:** Georgina Nakafero, Tricia McKeever, Niraj S Kukreja, Victoria Welsh, Sara Muller, Abhishek Abhishek

**Affiliations:** 1 Academic Rheumatology, University of Nottingham, Nottingham, UK; 2 School of Medicine, University of Nottingham, Nottingham, UK; 3 NIHR Nottingham Biomedical Research Centre, Nottingham, UK; 4 School of Medicine, Keele University, Newcastle-under-Lyme, UK; 5 Primary Care Centre Versus Arthritis, School of Medicine, Keele University, Newcastle-under-Lyme, UK

**Keywords:** Rheumatology, Epidemiology

## Abstract

**Objectives:**

To evaluate the association between fragility fracture and interruption of bisphosphonate prescription after three and five years of initial prescription, and to explore the incidence of atypical femoral fracture and osteonecrosis of jaw after three and five years of continued prescription of bisphosphonates.

**Design:**

Nested case-control and cohort studies.

**Setting:**

Clinical Practice Research Datalink Aurum, an anonymised longitudinal database of NHS primary care electronic health records, linked to hospital admission and mortality records from Hospital Episode Statistics and Office for National Statistics databases, respectively, 1 January 1997 to 31 December 2022.

**Participants:**

Adults aged ≥18 years on the date of the first prescription of alendronate or risedronate, and with primary care data linked to Hospital Episode Statistics and Office for National Statistics databases. Participants with a medication possession ratio of ≥67% in each of the first three and five years were followed up from the three or five year time points to the earliest of fragility fracture, death, end of study, two years, or last data availability. Cases were those who had a fragility fracture and were matched with up to four control participants.

**Main outcome measures:**

Incidence of fragility fracture, atypical femoral fracture, and osteonecrosis of jaw were main outcomes.

**Results:**

Data for 26 809 and 13 408 participants prescribed bisphosphonates for three and five years, respectively, were included. Fragility fracture was not associated with interruption of bisphosphonate prescription or intermittent prescription compared with continuous prescription in those previously prescribed bisphosphonates for three years (adjusted odds ratio 1.02, 95% confidence interval (CI) 0.85 to 1.24 and 1.13, 0.93 to 1.38, respectively) or five years (adjusted odds ratio 0.92, 0.75 to 1.13 and 0.87, 0.67 to 1.13, respectively). Time since last prescription was not associated with fragility fracture. After three and five years of bisphosphonate prescription, the incidence of atypical femoral fractures was 1.93 (95% CI 1.55 to 2.41) and 2.50 (1.92 to 3.26) per 1000 person years, respectively, and the incidence of osteonecrosis of jaw was 0.07 (0.02 to 0.21) and 0.00 per 1000 person years, respectively, within two years.

**Conclusion:**

Fragility fracture was not associated with interruption of bisphosphonate prescription for up to two years, after either three or five years of prescription. The incidence of atypical femoral fracture or osteonecrosis of jaw was uncommon and rare respectively.

**WHAT IS ALREADY KNOWN ON THIS TOPIC**

- Oral bisphosphonates reduce the risk of fracture, but long term use is associated with rare atypical femoral fracture and osteonecrosis of jaw, prompting guidance to consider treatment interruption (or drug holiday)
- Evidence supporting drug holidays is limited, with small extension trials and heterogeneous observational studies
- Signals for increased risk of fracture with longer interruptions of bisphosphonates are inconsistent

**WHAT THIS STUDY ADDS**

- In UK cohorts prescribed oral bisphosphonates for three or five years with a medication possession ratio of ≥67% in each year, interrupting prescription for up to two years was not associated with an increased odds of fragility fracture across a range of analytical approaches
- Atypical femoral fracture and osteonecrosis of jaw were uncommon and rare, respectively

**HOW THIS STUDY MIGHT AFFECT RESEARCH, PRACTICE OR POLICY**

- The findings support drug holidays for up to two years after bisphosphonate prescription for three or five years with good adherence
- Further research is needed to determine if patients at high risk of fragility fracture can also interrupt bisphosphonate treatment safely

## Introduction

 Bisphosphonates have been used in the management of osteoporosis for more than 30 years. Bisphosphonates have a persistent antiresorptive effect when treatment is discontinued after 3-5 years.^1^,^3^ The long term use of these drugs has been associated with atypical femoral fracture and osteonecrosis of jaw.^4 5^ To maximise patient benefit and minimise harm, treatment interruption is recommended. The Food and Drugs Administration, American Society for Bone and Mineral Research, and National Osteoporosis Guideline Group recommend bisphosphonate treatment for an initial period of 3-5 years in those at low risk, with prolonged treatment for 10 years recommended in those at high risk of fragility fracture.^6^,^9^ According to these guidelines, interrupting treatment for 1-3 years may be considered in those at low risk of fragility fracture. Clinical trials supporting the recommendations for treatment interruption (or drug holiday), however, only included 2496 participants in total.^1^,^3^

Interrupting treatment may be associated with an increased risk of fragility fractures. For example, withdrawal of risedronate for one year^10^ and alendronate for 2-3 years was associated with a reduction in bone mineral density.^1 11^ An increased risk of clinically apparent vertebral fractures was also found with alendronate withdrawal for 2-3 years.^1^ Similarly, withdrawal of zoledronate treatment was associated with an increased risk of morphometric vertebral fracture.^2^ A systematic review reported a higher risk of new clinical fractures of 20-40%, and the risk of vertebral fractures was approximately doubled with interruption of bisphosphonate treatment.^12^

To determine the duration of bisphosphonate prescription before interruption of treatment may be considered, and the risks of excess fragility fractures with different durations of drug holidays, we evaluated the association between fragility fracture and interrupting bisphosphonate prescription for one and two years in adults who were previously prescribed oral bisphosphonates for three and five years, respectively. We also explored the rates of atypical femoral fracture and osteonecrosis of jaw in adults after three and five years of bisphosphonate prescription.

## Methods

### Data source

The Clinical Practice Research Datalink Aurum^13^ is an anonymised longitudinal database of NHS primary care electronic health records that originated during routine clinical care. The Clinical Practice Research Datalink Aurum includes information on personal characteristics and lifestyle factors, diagnoses, test results, and primary care prescriptions. With universal coverage of English residents in the NHS, and healthcare being free at the point of delivery, participants who contribute data to the Clinical Practice Research Datalink are representative of the English population.^13^ Data in the Clinical Practice Research Datalink are linked to hospital admission and mortality records by linkages with Hospital Episode Statistics and Office for National Statistics databases, respectively.

### Study design and population

**Figure 1:**
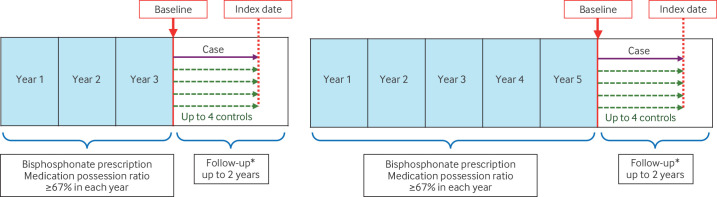
Schematic representation for establishing the study cohort over three (left) and five (right) years, with follow-up for up to two years to ascertain cases with fragility fractures. Cases were matched with up to four controls. *Follow-up is the period during which the use of bisphosphonate was assessed

Nested case-control and cohort studies examined the association between fragility fracture and bisphosphonate prescription status, and the incidence of atypical femoral fracture and osteonecrosis of jaw, respectively, from 1 January 1997 to 31 December 2022. The study population was adults aged ≥18 years on the date of first prescription of either alendronate or risedronate and with primary care data linked to Hospital Episode Statistics and Office for National Statistics databases (figure 1).

Only participants prescribed alendronate or risedronate were included because these drugs are currently the most prescribed oral bisphosphonates in the UK.^14^ Patients prescribed other antiresorptive or anabolic drugs before alendronate or risedronate, or prescribed bisphosphonates at age <18 years, before 1 January 1997, or within a year of registration in their current primary care doctor’s practice were excluded. Excluding patients registered for <1 year at their doctor’s practice minimised the risk of prevalent bisphosphonate users, poorly adherent to treatment, appearing as new users at a new doctor’s practice.

### Cohort establishment

Two separate cohorts were established. Participants prescribed bisphosphonates with a medication possession ratio of ≥67% in each of the first three and five years of prescription were ascertained (figure 1). The threshold for the medication possession ratio was informed by previous data on prescription refill that reduced the risk of fragility fracture.^15^ We chose a slightly higher medication possession ratio (≥67%) to define continued prescription than the 50% refill compliance (ie, collection of medication) that was reported to be associated with fewer fragility fractures.^15^ We derived the medication possession ratio from prescription data, regardless of whether the medicine was collected, because this information was not available in the Clinical Practice Research Datalink. We anticipated a small proportion of patients not collecting their prescriptions and therefore chose a slightly higher cut-off value for the medication possession ratio. Participants in the two cohorts were followed up from completion of three and five years of bisphosphonate prescription to the earliest date of outcome (ie, fragility fracture), death, end of the study, transfer out of their doctor’s practice, last data collection from the doctor’s practice, or at two years.

### Case-control definition

Cases were those who had a fragility fracture (ie, hip, wrist, vertebral, or osteoporotic (fragility) fracture with site unspecified) recorded in primary care, hospital admission, or mortality records within two years after three and five years of bisphosphonate prescription. We used a two year follow-up period because a two year interruption in treatment was associated with a reduction in bone mineral density and clinically apparent vertebral fracture.^1 10 11^ Fragility fracture was determined in all data sources to allow for complete capture of events. If we found more than one record during follow-up, we considered the earliest record in this period. Up to four participants without a fragility fracture after three and five years of bisphosphonate prescription were matched to each case for age at the start of follow-up (±2 years), sex, and year of start of follow-up, with risk set matching. Data for sex were taken from information in the Clinical Practice Research Datalink Aurum rather than from patient reported gender.

The date of fragility fracture was the index date for cases. The date on which a case had a fragility fracture during follow-up was allocated as the index date for its matched controls. This approach ensured comparable follow-up time to accrue bisphosphonate prescription between cases and matched controls.

### Prescription of bisphosphonates

Prescription of bisphosphonates was assessed between the start of follow-up (ie, three or five years from the first prescription date) and the index date. Medication possession ratios in this period were used to define prescription status as prescription interruption (medication possession ratio ≤33%), intermittent prescription (medication possession ratio 34-66%), and continued prescription (medication possession ratio ≥67%). Medication possession ratio ≤33% was considered a reasonable surrogate for treatment interruption because almost no reduction in the risk of fracture exists at this level of bisphosphonate prescription.^15^ Fracture risk reduces at a refill rate of >50%.^15^ To minimise any misclassification from prescriptions that were not collected, however, continued prescription was defined as a medication possession ratio of ≥67%.^15^ Time since last bisphosphonate prescription at the index date and cumulative number of days of bisphosphonate prescription between the start of follow-up and the index date were the other variables of interest.

### Positive and negative control variables

Opioid prescription, categorised as recent (≥1 prescription within 90 days of the index date), remote (≥1 prescription within 91-365 days of the index date), and past or no prescription, was a positive control variable because of its association with fragility fracture.^16^ A previous diagnosis of osteoarthritis (yes, no) was a negative control variable because of its lack of association with fragility fractures.^17^

### Determining atypical femoral fracture or osteonecrosis of jaw

Patients included in the cohorts who did not have an atypical femoral fracture or osteonecrosis of jaw before the start of follow-up were followed up to the earliest of outcome (ie, atypical femoral fracture or osteonecrosis of jaw, as appropriate), date of the last bisphosphonate prescription, end of the study, death, two years, or last data availability. Atypical femoral fracture was defined as present if we found a primary care, hospital admission, or mortality record. Osteonecrosis of jaw was established with the two step algorithm from Persson et al, with a positive predictive value of 92%.^5^ We first identified potential osteonecrosis from primary care or hospital admission records. We then distinguished osteonecrosis of jaw from other osteonecrosis by identifying confirmatory evidence of jaw involvement; strong codes (ie, those explicitly indicating osteonecrosis of jaw) within one year or weak codes (jaw specific conditions or procedures plausibly consistent with osteonecrosis of jaw) within 90 days. Cases were defined as having osteonecrosis of jaw when the osteonecrosis code co-occurred with any evidence of jaw involvement. The date of the first record of atypical femoral fracture or osteonecrosis of jaw in any dataset was the outcome date.

### Statistical analyses

In nested case-control studies, multivariable conditional logistic regression was used to assess the association between fragility fracture and interruption of bisphosphonate prescription and cumulative bisphosphonate prescription. Association with time since last bisphosphonate prescription was estimated from a conditional logistic regression model with restricted cubic splines.

Two models were constructed. Model 1 was adjusted for matching variables. Model 2 was additionally adjusted for body mass index, ethnic group, smoking status (current smoker, ex-smoker, or non-smoker), alcohol intake (low (<14 units/week), moderate (14-21 units/week), high (>21 units/week), previous alcohol intake, or no alcohol intake), nomenclature of territorial units for statistics level 1 geographical region, individual index of multiple deprivation (categorised as five equal groups), Charlson comorbidity index, autoimmune rheumatic disease (yes/no), inflammatory bowel disease (yes/no), chronic kidney disease stage ≥3 (yes/no), vitamin D with or without a calcium prescription in the previous 90 days, corticosteroids prescription in the previous 90 days, and number of primary care consultations and hospital admissions in the 12 months before the index date.

Fractional polynomials were used to model a non-linear relation between fragility fracture and body mass index before these variables were considered in the model.^18^ Multiple imputation with chained equations was used to impute missing data for body mass index, ethnic group, deprivation, and smoking and alcohol consumption, with the Stata command mi impute. The imputation model included all listed covariates, bisphosphonate medication possession ratio during follow-up, and outcome. We calculated and combined adjusted odd ratios and 95% confidence intervals (CIs) with Rubin's rule across 20 imputations.

We tested for interactions between prespecified prognostic factors: age (≤70 *v* >70 years), sex (women or men), deprivation (first two *v* last three groups), ethnic group (white or non-white), drug (alendronate or risedronate), previous fragility fracture, previous hip fracture, and bisphosphonate prescription status up to two years before the index date in the fully adjusted model. About 95% of the study population were white participants, with few people from many other ethnic groups. The small number of participants from diverse ethnicities restricted us to explore ethnic group as a two level variable (white and non-white). Stratified analyses were planned if evidence of statistical interaction existed. Stratified analyses were presented for sex after reviewer comments.

We undertook several sensitivity analyses. Firstly, we required all participants in the nested case-control study to have a full one year period immediately before the index date in which bisphosphonate prescription status could be determined. The minimum one year follow-up time before index date allowed us to look at any potential bias caused by the medication possession ratio being artificially high in patients who had a fragility fracture soon after the start of the follow-up period. Secondly, we defined continued treatment as a medication possession ratio ≥80%, a widely accepted threshold for optimal medication possession. Thirdly, we used different thresholds for the medication possession ratio (ie, 0-24%, 25-49%, 50-74%, and ≥75%). Finally, we considered only hip fracture and vertebral fracture to define case status, allowing us to explore if treatment interruption could increase the risk of more severe outcomes.

For assessment of bias and residual confounding, we undertook quantitative bias analysis for misclassification of bisphosphonate prescription status during follow-up.^19^ An E value was calculated to assess how strong unmeasured confounding would have to be to negate the observed results of the main analysis.^20^ We explored the association between negative and positive control variables and outcomes with multivariable conditional logistic regression.

For the cohort study, we calculated the incidence rate (95% CI) of atypical femoral fracture and osteonecrosis of jaw per 1000 person years at different follow-up times. The rate was grouped according to age (>65, 65-79, or ≥80 years), sex (men or women), ethnic group (white or non-white), bisphosphonate (alendronate or risedronate), number of glucocorticoid prescriptions in the 12 months before the start of follow-up, and previous fragility fracture. We also calculated the incidence at one, five, and 10 years.

### Patient and public involvement

The study used pseudoanonymised data, and thus study participants could not be contacted for involvement in this research. We plan to engage the public and stakeholders on our findings by dissemination through presentation at conferences and through social media. Working with patient and public involvement members, we will produce a lay summary, infographic, and a video, and disseminate these through the Royal Osteoporosis Society and Arthritis UK.

## Results

**Figure 2:**
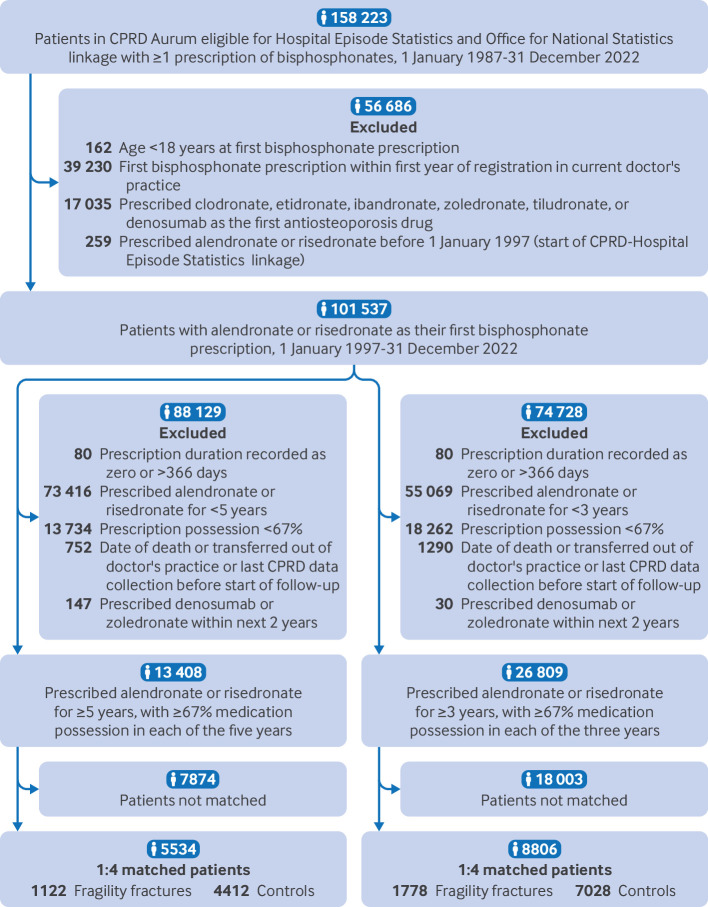
Study population selection criteria. CPRD=Clinical Practice Research Datalink

Data for 26 809 and 13 408 participants prescribed bisphosphonates for three and five years, respectively, were included (figure 2).

### Fragility fracture and bisphosphonate prescription interruption

We identified 8806 participants (1778 with fragility fractures and 7028 matched controls without fragility fractures) and 5534 participants (1122 with fragility fractures and 4412 matched controls without fragility fractures) in the nested case-control studies that evaluated the association between fragility fractures and interruption in bisphosphonate prescription after three and five years of prescription, respectively (figure 2). In both case-control studies, cases were more often of the white ethnic group, belonged to the most deprived group based on the index of multiple deprivation, were current smokers, had hazardous alcohol use, a higher Charlson comorbidity index, and more hospital admissions and primary care consultations than controls (table 1).

**Table 1:** Characteristics of the population included in the nested case-control studies*

Covariates	Bisphosphonate prescription cohort			
	3 year prescription		5 year prescription	
	Controls (n=7028)	Cases (n=1778)	Controls (n=4412)	Cases (n=1122)
Mean (SD) age (years)	79 (10)	80 (10)	80 (9)	80 (9)
Mean (SD) body mass index	25.7 (5.2)	24.9 (5.2)	25.7 (5.19)	25.0 (5.4)
Missing body mass index value	403 (5.7)	117 (6.6)	198 (4.5)	43 (3.9)
Sex:				
Men	962 (13.7)	248 (14)	573 (13.0)	152 (13.5)
Women	6066 (86.3)	1530 (86.1)	3839 (87.0)	970 (86.5)
Ethnic group:				
White	6524 (92.8)	1696 (95.4)	4102 (93.0)	1077 (96)
Mixed	119 (1.7)	16 (0.9)	78 (1.8)	13 (1.2)
Bangladeshi, Indian, or Pakistani	92 (1.3)	20 (1.1)	58 (1.3)	8 (0.7)
Black	71 (1.0)	10 (0.6)	48 (1.1)	-
Chinese or other Asian	52 (0.7)	5 (0.3)	29 (0.7)	-
Other	55 (0.9)	9 (0.5)	37 (0.8)	9 (0.8)
Missing	115 (1.6)	22 (1.2)	60 (1.4)	7 (0.6)
Geographical region:				
Northeast	432 (6.2)	116 (6.5)	291 (6.6)	77 (6.9)
Northwest	249 (3.5)	56 (3.2)	131 (3.0)	37 (3.3)
Yorkshire and The Humber	525 (7.5)	173 (9.7)	369 (8.4)	107 (9.5)
East Midlands	325 (4.6)	85 (4.8)	194 (4.4)	51 (4.6)
East of England	711 (10.1)	153 (8.6)	445 (10.1)	107 (9.5)
London	961 (13.7)	217 (12.2)	628 (14.2)	149 (13.3)
Southeast	2310 (32.9)	534 (30.0)	1394 (31.6)	337 (30.0)
Southwest	1515 (21.6)	444 (25.0)	960 (21.8)	257 (22.9)
Index of multiple deprivation (group):				
1 (least deprived)	2124 (30.2)	499 (28.1)	1379 (31.3)	310 (27.6)
2	1592 (22.7)	380 (21.4)	978 (22.2)	238 (21.2)
3	1351 (19.2)	354 (19.9)	839 (19.0)	232 (20.7)
4	1165 (16.6)	303 (17.0)	723 (16.4)	184 (16.4)
5 (most deprived)	793 (11.3)	240 (13.5)	490 (11.1)	157 (14.0)
Missing	3 (0.04)	2 (0.1)	3 (0.1)	1 (0.1)
Smoking status:				
Non-smoker	4527 (64.4)	1073 (60.4)	2862 (64.9)	675 (60.2)
Current smoker	445 (6.3)	164 (9.2)	267 (6.1)	100 (8.9)
Ex-smoker	2016 (28.7)	533 (30.0)	1277 (28.9)	344 (30.7)
Missing	40 (0.6)	8 (0.5)	6 (0.1)	3 (0.3)
Alcohol consumption (units/week):				
Non-drinker	1430 (20.4)	357 (20.1)	938 (21.3)	231 (20.6)
Low (1-14)	2912 (41.4)	689 (38.8)	1841 (41.7)	433 (38.6)
Moderate (15-21)	168 (2.4)	48 (2.7)	98 (2.2)	36 (3.2)
Hazardous (>21)	239 (3.4)	99 (5.6)	160 (3.6)	69 (6.2)
Former drinker	901 (12.8)	236 (13.3)	595 (13.5)	159 (14.2)
Missing	1378 (19.6)	349 (19.6)	780 (17.7)	194 (17.3)
Diseases:				
Autoimmune rheumatic diseases†	1558 (22.2)	322 (18.1)	933 (21.2)	224 (20.0)
Inflammatory bowel disease	139 (1.9)	28 (1.6)	89 (2.0)	28 (2.5)
Chronic kidney disease (stage 3)	1706 (24.3)	432 (24.3)	1178 (26.7)	306 (27.3)
Charlson's comorbidity index:				
0	1820 (25.9)	422 (23.7)	1194 (27.1)	258 (23.0)
1	1263 (18.0)	301 (16.9)	709 (16.1)	204 (18.2)
2	1405 (20.0)	383 (21.5)	877 (20.0)	199 (17.7)
3	1003 (14.3)	254 (14.3)	620 (14.1)	173 (15.4)
≥4	1537 (21.9)	418 (23.5)	1012 (22.9)	288 (25.7)
No of hospital admissions:‡				
0	4777 (68.0)	830 (46.7)	3099 (70.2)	505 (45.0)
1	1325 (18.9)	463 (26.0)	762 (17.3)	336 (30.0)
2	501 (7.1)	216 (12.2)	310 (7.0)	131 (11.7)
≥3	425 (6.1)	269 (15.1)	241 (5.5)	150 (13.4)
No of primary care consultations (divided by quartiles):‡				
1	1937 (27.6)	372 (30.0)	1248 (28.3)	188 (16.8)
2	1950 (27.8)	399 (22.4)	1092 (24.8)	246 (22.0)
3	1652 (23.5)	403 (22.7)	1117 (25.3)	289 (25.8)
4	1489 (21.2)	604 (34.0)	955 (21.7)	399 (35.6)
Drugs:				
Vitamin D with or without calcium in previous 90 days	4641 (66.0)	1178 (66.3)	2923 (66.3)	764 (68.1)
Corticosteroid prescriptions in previous 90 days	1085 (15.4)	239 (13.4)	590 (13.4)	155 (13.8)

Data are number (%) unless indicated otherwise.

Data suppressed because value <5 in at least one category

* Characteristics were determined on the index date. Index date was defined as fracture date for cases and as start of follow-up together with time until fracture date for matched controls; the latest record before the index date was considered for personal characteristics, lifestyle factors, and comorbidities.

† Includes rheumatoid arthritis, psoriatic arthritis, ankylosing spondylitis, giant cell arteritis, vasculitis, polymyalgia rheumatica, reactive arthritis, systemic lupus erythematosus, and connective tissue diseases.

‡ Within 12 months before the index date.

SD, standard deviation.

Fragility fracture was not associated with interruption of bisphosphonate prescription and intermittent prescription within two years before the index date, with adjusted odds ratio of 1.02 (95% CI 0.85 to 1.24) and 1.13 (0.93 to 1.38), respectively, compared with continued prescription in the case-control study nested in the cohort prescribed bisphosphonates for three years (table 2). We found no association between fragility fracture and cumulative bisphosphonate prescription. Similar findings were seen in the cohort prescribed bisphosphonates for five years for both bisphosphonate prescription interruption and intermittent prescription (table 2). Results from a fully adjusted model that included previous fracture in the cohort prescribed bisphosphonates for three years showed an adjusted odds ratio of 1.15 (95% CI 0.94 to 1.41) for intermittent prescription and adjusted odds ratio of 1.03 (0.85 to 1.25) for interruption of bisphosphonate prescription; for the cohort prescribed bisphosphonates for five years, the values were adjusted odds ratio 0.84 (95% CI 0.64 to 1.09) for intermittent prescription and adjusted odds ratio 0.88 (0.71 to 1.08) for interruption of bisphosphonate prescription.

Results were similar in men and women (online supplemental table S1). Increasing time since last bisphosphonate prescription was not associated with fragility fracture in the cohort prescribed bisphosphonates for three years (online supplemental figure S1) or five years (online supplemental figure S2).

**Table 2:** Association between fragility fractures and oral bisphosphonate prescription interruption: nested case-control study

Duration of follow-up	Previous 3 years of bisphosphonate prescription				Previous 5 years of bisphosphonate prescription			
	Controls (No (%))	Cases (No (%))	Model 1 odds ratio (95% CI)	Model 2 odds ratio (95% CI)	Controls (No (%))	Cases (No (%))	Model 1 odds ratio (95% CI)	Model 2 odds ratio (95% CI)
Up to 24 months:								
Continuous prescription (≥67%)	5669 (80.7)	1382 (77.7)	1	1	3286 (74.5)	848 (75.6)	1	1
Intermittent prescription (34-66%)	521 (7.4)	164 (9.2)	1.29 (1.07 to 1.55)	1.13 (0.93 to 1.38)	363 (8.2)	86 (7.7)	0.91 (0.71 to 1.17)	0.87 (0.67 to 1.13)
Prescription interruption (0-33%)	838 (11.9)	232 (13.1)	1.17 (0.98 to 1.40)	1.02 (0.85 to 1.24)	763 (17.3)	188 (16.8)	0.96 (0.79 to 1.17)	0.92 (0.75 to 1.13)
Cumulative duration of bisphosphonate prescription before index date (days)*								
Group 4 (478-732)	1757 (25.0)	443 (24.9)	1	1	1081 (24.5)	279 (24.9)	1	1
Group 3 (282-476)	1538 (21.9)	390 (21.9)	1.01 (0.83 to 1.23)	0.96 (0.78 to 1.17)	1011 (22.9)	259 (23.1)	0.99 (0.78 to 1.27)	0.97 (0.75 to 1.26)
Group 2 (114-280)	1708 (24.3)	411 (23.1)	0.97 (0.78 to 1.20)	0.90 (0.72 to 1.13)	1208 (27.4)	311 (27.7)	0.99 (0.76 to 1.28)	0.97 (0.73 to 1.27)
Group 1 (0-112)	2025 (28.8)	534 (30.0)	1.16 (0.92 to 1.46)	0.97 (0.76 to 1.24)	1112 (25.2)	273 (24.3)	0.92 (0.70 to 1.21)	0.86 (0.64 to 1.15)
Up to 12 months:								
Continuous prescription (≥67%)	3091 (77.9)	754 (76.7)	1	1	1980 (74.0)	498 (73.3)	1	1
Intermittent prescription (34-66%)	276 (7.0)	73 (7.4)	1.10 (0.83 to 1.44)	0.92 (0.69 to 1.23)	181 (6.8)	54 (8.0)	1.17 (0.85 to 1.62)	1.13 (0.80 to 1.60)
Prescription interruption (0-33%)	601 (15.2)	156 (15.9)	1.08 (0.86 to 1.36)	0.95 (0.75 to 1.21)	514 (19.2)	127 (18.7)	0.99 (0.77 to 1.26)	0.98 (0.75 to 1.27)
Cumulative duration of bisphosphonate prescription before index date (days)*								
Group 4 (253-366)	885 (22.3)	229 (23.3)	1	1	663 (24.8)	164 (24.2)	1	1
Group 3 (141-252)	1028 (25.9)	225 (22.9)	0.77 (0.59 to 1.00)	0.77 (0.59 to 1.02)	646 (24.2)	185 (27.3)	1.19 (0.88 to 1.59)	1.25 (0.91 to 1.71)
Group 2 (57-140)	890 (22.4)	219 (22.3)	0.95 (0.71 to 1.29)	0.88 (0.64 to 1.20)	497 (18.6)	108 (15.9)	0.82 (0.57 to 1.18)	0.87 (0.59 to 1.28)
Group 1 (0-56)	1165 (29.4)	310 (31.5)	1.13 (0.83 to 1.56)	0.91 (0.65 to 1.28)	869 (32.5)	222 (32.7)	0.99 (0.70 to 1.40)	0.97 (0.68 to 1.40)

Model 1 adjusted for matching variables (age at start of follow-up, sex, and year at start of follow-up). Model 2 adjusted for age at start of follow-up, sex, year at start of follow-up, body mass index, ethnic group, region, deprivation, smoking, alcohol intake, autoimmune rheumatic diseases, inflammatory bowel disease, chronic kidney disease stage 3, Charlson comorbidity index, number of primary care consultations in 12 months before index date, number of hospital admissions in 12 months before index date, vitamin D with or without calcium supplement or treatment, and corticosteroids in previous 90 days of index date.

* In the previous three years treatment cohort. Minimum and maximum cumulative duration of bisphosphonate treatment before the index date in the previous five years of treatment cohort in each group are as follows: for up to 24 months: group 4 (424-732 days), group 3 (225-420 days), group 2 (72-224 days), and group 1 (0-70 days); for up to 12 months: group 4 (225-366 days), group 3 (119-224 days), group 2 (57-116 days), and group 1 (0-56 days).

CI, confidence interval.

Tests for interaction between the bisphosphonate medication possession ratio during follow-up and sex, deprivation, ethnic group, drug, previous fragility fractures, and previous hip fractures on fragility fracture were not significant in the cohort prescribed bisphosphonates for three years, with P values of 0.79, 0.94, 0.83, 0.30, 0.85, 0.48, and 0.17, respectively. In the cohort prescribed bisphosphonates for five years, P values were 0.22, 0.86, 0.90, 0.63, 0.56, 0.85, and 0.63, respectively.

### Sensitivity analyses

Findings were similar to the main analysis when different levels of medication possession ratio were considered to define prescription status, and on considering interruption of bisphosphonate prescription within the 12 month period before the index date in both nested case-control studies (online supplemental tables S2-S4). Hip fracture was not associated with interruption of bisphosphonate prescription (adjusted odds ratio 1.06, 95% CI 0.73 to 1.53) but was associated with intermittent prescription (adjusted odds ratio 1.47, 1.00 to 2.16) within two years before the index date compared with continued prescription in the case-control study nested in the cohort prescribed bisphosphonates for three years (online supplemental table S5). No association, however, was found in the case-control study nested in the cohort prescribed bisphosphonates for five years (online supplemental table S5). We found no association between interruption of bisphosphonates and increased risk of hip fracture in women or men in both nested case-control studies (online supplemental table S5). We also found no association between interruption of bisphosphonate prescription or intermittent prescription and vertebral fractures in both nested case-control studies (online supplemental table S6).

### Quantitative bias analysis

In a cohort prescribed bisphosphonates with high levels of medication possession for three (or five) years, we would not anticipate a switch to a second line osteoporosis drug treatment without an intercurrent event (ie, fragility fracture). Similarly, we would not expect many patients to have side effects from bisphosphonates during established treatment and to then change treatment. We anticipated that most patients in the three (or five) year cohorts would not be affected by misclassification of drug prescription, and no difference between cases and controls was expected in this regard. Nevertheless, assuming a minimum of 90% sensitivity and 90% specificity, and trapezoidal distribution, the univariate odds ratio accounting for such levels of misclassification would be 1.22 (95% CI 0.97 to 1.66) in the three year cohort and 0.94 (0.73 to 1.18) in the five year cohort for interruption versus continued treatment.

### Assessment of residual confounding

E values were 1.51 and 1.16 for intermittent prescription and prescription interruption, respectively, in the three year cohort, and 1.56 and 1.39 for intermittent prescription and prescription interruption, respectively, in the five year cohort. A prescription of opioids within the previous 90 days was associated with an increased risk of fragility fracture, whereas a diagnosis of osteoarthritis was not associated with fragility fracture (online supplemental table S7).

### Incidence of atypical femoral fracture

After three and five years of bisphosphonate prescription, 78 and 55 atypical femoral fractures occurred within the next two years at an incidence rate of 1.93 (95% CI 1.55 to 2.41) and 2.50 (1.92 to 3.26) per 1000 person years, respectively. We found similar incidence rates during the first year, but the incidence rates were higher when the follow-up period was up to five and 10 years (table 3). When those with a record of atypical femoral fracture before the start of follow-up were included, the incidence rate of atypical femoral fracture was only marginally higher (online supplemental table S8).

**Table 3:** Incidence rate of atypical femoral fractures in patients prescribed oral bisphosphonates

Characteristics	Previous 3 years of bisphosphonate prescription			Previous 5 years of bisphosphonate prescription		
	No of outcomes	Person years	Incidence rate (95% CI)/1000 person years	Outcomes (n)	Person years	Incidence rate (95% CI)/1000 person years
Overall incidence over 0-2 years	78	40 381	1.93 (1.55 to 2.41)	55	21 984	2.50 (1.92 to 3.26)
Grouped analyses						
Age (years):						
<65*	6	7396	0.81 (0.36 to 1.81)	—	—	1.22 (0.46 to 3.24)
65-79	29	18 391	1.58 (1.10 to 2.27)	22	10 357	2.12 (1.40 to 3.23)
≥80	43	14 593	2.95 (2.19 to 3.97)	29	8339	3.48 (2.42 to 5.00)
Sex:						
Men	10	7445	1.34 (0.72 to 2.50)	8	3727	2.15 (1.07 to 4.29)
Women	68	32 936	2.06 (1.63 to 2.62)	47	18 257	2.57 (1.93 to 3.43)
Ethnic group:						
Not white*	—	—	0.26 (0.04 to 1.83)	—	—	1.38 (0.45 to 4.29)
White	-	-	2.11 (1.69 to 2.64)	-	-	2.62 (2.00 to 3.44)
Type of bisphosphonate:						
Alendronate	63	34 780	1.81 (1.42 to 2.32)	46	18 762	2.45 (1.84 to 3.27)
Risedronate	15	5601	2.68 (1.61 to 4.44)	9	3222	2.79 (1.45 to 5.37)
No of steroid prescriptions†:						
0	64	30 730	2.08 (1.63 to 2.66)	41	17 322	2.37 (1.74 to 3.21)
1- 5	6	3918	1.53 (0.69 to 4.41)	8	1954	4.09 (2.05 to 8.19)
≥6	8	5733	1.40 (0.70 to 2.79)	6	2709	2.22 (1.00 to 4.93)
Previous fragility fracture:						
No	30	23 574	1.27 (0.89 to 1.82)	21	12 895	1.63 (1.06 to 2.50)
Yes	48	16 807	2.86 (2.15 to 3.79)	34	9089	3.74 (2.67 to 5.24)
Rate with different follow-up times (years):						
0-1	42	22 711	1.85 (1.37 to 2.50)	28	12 344	2.27 (1.57 to 3.29)
0-5	182	74 075	2.46 (2.12 to 2.84)	111	40 439	2.74 (2.28 to 3.31)
0-10	277	97 109	2.85 (2.54 to 3.21)	159	52 377	3.04 (2.60 to 3.55)

* Events and person time suppressed because of <5 outcomes in at-least one sensitive category.

† In 12 months before start of follow-up.

CI, confidence interval.

The incidence of atypical femoral fracture was higher in those aged ≥80 years and with previous fragility fractures (table 3). In the cohort prescribed bisphosphonates for three years, compared with patients without atypical femoral fracture, patients with atypical femoral fracture were more often women (81% *v* 85.9%), aged ≥80 years (38.2% *v* 54.1%), underweight (4.1% *v* 10.6%), current smokers (8.7% *v* 10.6%), white ethnic group (90.1 *v* 98.8%), prescribed risedronate (13.5 *v* 21.2%), and had a previous fragility fracture (43.5% *v* 64.7%) (online supplemental table S9). These findings were broadly similar in the cohort prescribed bisphosphonates for five years (online supplemental table S9).

### Incidence of osteonecrosis of jaw

After three years of bisphosphonate prescription, <5 (most established from hospital records, and all prescribed alendronate) cases of osteonecrosis of jaw were identified within the next two years at a rate of 0.07 (95% CI 0.02 to 0.21) per 1000 person years. After five years of bisphosphonate prescription, no cases of osteonecrosis of jaw occurred within the next two years. With alternative follow-up windows, incidence rates for the three year bisphosphonate prescribed cohort were 0.04 (95% CI 0.01 to 0.29) at one year, 0.10 (0.05 to 0.19) at five years, and 0.17 (0.12 to 0.26) at 10 years; incidence rates for the five year bisphosphonate prescribed cohort were 0.00 at one year, 0.10 (0.04 to 0.24) at five years, and 0.25 (0.16 to 0.40) per 1000 person years at 10 years. Most cases of osteonecrosis of jaw identified in this study (78.3% in three year cohort and 77.8% in five year cohort) had strong codes, as defined by Persson et al.^5^ When those with a recorded outcome before the start of follow-up were included, the incidence of osteonecrosis of jaw was marginally higher (online supplemental table S8). The small numbers of patients with osteonecrosis of jaw did not allow a meaningful comparison of the characteristics of patients with or without osteonecrosis of jaw.

## Discussion

### Principal findings

In this nationally representative cohort of people prescribed bisphosphonates for three and five years with a ≥67% medication possession ratio in each year, we found no evidence of an increase in the odds of fragility fractures within the next two years when bisphosphonate prescription was discontinued, or if prescription was infrequent. The findings were robust to misclassification of bisphosphonate prescription during follow-up and no evidence of residual confounding was found. Nevertheless, these findings should be interpreted with caution because the study design was observational. Patients included in the nested case-control studies had a high medication possession ratio in the run-in phase. These findings should not be extrapolated to those with lower levels of medication possession because these individuals may still have an increased risk of fragility fractures.

The five year cohort was a subset of the three year cohort, reflecting real world clinical practice where patients who have been treated for five years would also have been treated for three years. The findings of the two nested case-control studies, however, were not completely independent. The use of bisphosphonates was averaged over two years (one year in a sensitivity analysis) which obscured temporary discontinuations. We do not think that the duration or timing of these breaks would have a substantial effect on the risk of fractures because individuals prescribed bisphosphonates for 3-5 years continue to have suppressed bone turnover markers for 1-2 years after treatment is discontinued.

We found an increase in the odds of hip fractures with intermittent prescription in the three year cohort but no association with a pause in treatment. This observation could have been a chance finding in a sensitivity analysis and should be confirmed in future studies.

### Comparison with other studies

Our findings are consistent with previous reports from the US.^21 22^ In these US studies, interruption of bisphosphonate for ≥1 year was not associated with fragility fractures or hip fractures in patients prescribed bisphosphonates for three years with a 50% medication possession ratio, or in patients prescribed bisphosphonates for five years with 60% proportion of days covered, respectively.^21 22^ These studies were restricted to women, whereas our study included both men and women and considered longer pauses in treatment. In a previous study based on Medicare data from the US, an increased risk of hip, clinically symptomatic vertebral, and humeral fractures was found with interruption in alendronate prescription for >2 years, and an increased risk of hip and clinical vertebral fractures with interruption in risedronate prescription for >2 years after treatment for three years with excellent adherence (medication possession ratio ≥80%).^23^ A recent systematic review reported an increase in bone turnover markers and reduction in bone mineral density during a bisphosphonate drug holiday after an initial 3-5 years of treatment.^24^ Our study, together with previous studies from the US,^21 22^ indicates that this change does not translate into an increase in the risk of clinical fracture for an interruption in bisphosphonate prescription of up to two years that is preceded by previous treatment for a minimum of three years with acceptable measures of adherence.

Our findings are not consistent with those from a nationwide Danish study that reported a significantly lower risk of hip fractures in patients prescribed alendronate for 5-10 years or >10 years than those prescribed bisphosphonates for <5 years.^25^ A key difference between the Danish study and our study was that we included patients who were adherent to bisphosphonates, with a ≥67% medication possession ratio, whereas the Danish study included naive alendronate users, and the population was not restricted to regular prescriptions.

We found no evidence of a significant interaction between the prescription of bisphosphonates and increasing age, previous fragility fracture, or previous hip fracture. The current guidance is for these patients to receive antiresorptive drug treatment for 10 years, and treatment interruption is not specifically recommended.^9^ Our findings raise the possibility that such patients could pause treatment for up to two years, perhaps after five years of bisphosphonate prescription, if adherence is good. This finding, however, should be confirmed in other studies before changing clinical practice. A pause in bisphosphonate treatment has been associated with a reduced risk of atypical femoral fracture.^4 26^ Future research should explore further the effect of discontinuing bisphosphonates on the risk of osteonecrosis of jaw.

Atypical femoral fracture and osteonecrosis of jaw were uncommon and rare side effects, respectively, in our study. We identified only a few incidences of osteonecrosis of jaw outcomes, resulting in imprecise estimates, and hence the incidence of osteonecrosis of jaw reported by us should be interpreted with caution. Our estimated rates of atypical femoral fracture were comparable with those reported in a retrospective cohort study of data from Kaiser Permanente Southern California.^4^ The increase in the rate of atypical femoral fracture observed in our study with increasing treatment duration, however, was not as steep.^4^ Our estimated rates of osteonecrosis of jaw were similar to those reported in a previous UK study, but marginally higher than those reported in Danish and Korean studies, possibly because we established incidences of osteonecrosis of jaw from both hospital admission and primary care records with a validated definition.^5 27 28^ Underestimation of the incidence rate in these studies could have occurred because stage 1 and stage 2 osteonecrosis of jaw are managed with optimising oral hygiene, broad spectrum antibiotics, and cessation of any offending drug, and do not require surgical treatment. Although concerns exist about the reliability of determining the incidence of osteonecrosis of jaw in electronic healthcare records,^29^ most of our cases were established from data in hospital discharge summaries. The higher rates of osteonecrosis of jaw in patients prescribed alendronate than risedronate. is consistent with a previous report from the French Pharmacovigilance Database.^30^

### Strengths and limitations of this study

One of the strengths of our study was the large nationwide database, representative of the general population, that included women and men across all age ranges. The use of data from primary care consultations and prescriptions, and hospital admission and mortality records allowed us to capture all clinically apparent fragility fractures from all possible data sources, thus minimising any bias from outcome misclassification. Almost all of the atypical femoral fracture and osteonecrosis of jaw outcomes were determined from hospital admission and mortality records. The positive predictive value of subtrochanteric femur or femoral shaft fracture, and subtrochanteric or diaphyseal femoral fractures recorded in hospital discharge summaries is reported to be 90-92%.^31 32^ We found <5 hospital admissions for road traffic incidents within 90 days before atypical femoral fractures (the exact number is not given because of disclosure rules in the Clinical Practice Research Datalink to prevent inadvertent identification of patients with rare outcomes), and almost all atypical femoral fractures could exclusively be attributed to the use of bisphosphonates. The very small number of atypical femoral fractures that could be attributed to another cause increases the validity of our findings. Large scale adjustment with covariates was conducted to minimise confounding. The associations were consistent across a series of clinically relevant sensitivity analyses. The results were robust to misclassification of bisphosphonate prescription during follow-up. We found no evidence of residual confounding when we analysed negative control variables, but residual confounding cannot be ruled out.

The study had several limitations. As an observational study, only association and not causation should be inferred. Also, although fragility fractures, osteonecrosis of jaw, and atypical femoral fracture were determined from general practice consultation, hospital admission, procedure, and mortality records, each outcome was not verified. This approach, however, has been widely used in osteoporosis research. We used a two stage algorithm with a positive predictive value of 92% for determining osteonecrosis of jaw to improve accuracy, and as outlined above, almost all atypical femoral fractures could be exclusively attributed to the use of bisphosphonates.

We did not have data for dual x ray absorptiometry scans, and therefore we could not consider bone mineral density, an indicator of the severity of osteoporosis, in our analyses. Similarly, the Clinical Practice Research Datalink does not have the results of radiological evaluations. Consequently, we could not include vertebral fractures that were only apparent on imaging as an outcome if not also coded in the medical record, and this could have introduced misclassification bias. Also, if individuals did not seek medical consultation for a fracture, these fractures were not included in the study. The date of the fracture recorded in the electronic healthcare records could have been later than the date on which the event occurred. This number of days is likely to be small, however, because fragility fractures are extremely painful. The study spanned 27 years, but patients and controls were matched for calendar year.

Patients who stopped bisphosphonate treatment after three and five years with a medication possession ratio ≥67% in each year could have been prescribed an alternate drug from a hospital clinic (eg, teriparatide, denosumab, or intravenous zoledronate). The absence of their recording in the Clinical Practice Research Datalink could potentially minimise any effect of interruption of bisphosphonate treatment on an increase in the risk of fracture. In a cohort of adults regularly prescribed oral bisphosphonates for at least three years, however, such treatment changes would typically occur after a fragility fracture, an outcome at which follow-up would cease. Also, the medication possession ratio, used to assess drug use status, could have overestimated actual drug consumption but any such bias is likely to be non-differential.

Data on tooth extraction were not available and the association between tooth extraction and osteonecrosis of jaw in patients prescribed bisphosphonates should be explored further. Also, we did not have data on the severity of previous fractures, physical activity, and most risk factors for falls because these data were not available in the Clinical Practice Research Datalink. We could not adjust for these data in our analyses, which is a key limitation of our study. We found no evidence, however, of residual confounding on negative control analysis. Finally, the 95% confidence intervals were broad and therefore smaller levels of increased risk of fragility fracture from treatment interruption would not have been detected.

### Conclusions

The results of our study suggested that interrupting bisphosphonate prescription for up to two years, after a minimum of three years of regular bisphosphonate prescription (medication possession ratio ≥67%) was not associated with an increased risk of fragility fractures. Increasing prescription duration was associated with infrequent but potentially serious side effects.

## Supplementary material

10.1136/bmjmed-2025-002085 online supplemental file 1

10.1136/bmjmed-2025-002085 online supplemental file 2

## Data Availability

Data may be obtained from a third party and are not publicly available.
