# Supplementary material for: Fragility fracture, atypical femoral fracture, and osteonecrosis of jaw after bisphosphonate prescription for three and five years, based on primary and secondary care data in England: nested case-control and cohort studies
Source: BMJ Med. 2026 Apr 22;5(1):e002085. doi: 10.1136/bmjmed-2025-002085 (PMC13110635; doi:10.1136/bmjmed-2025-002085)
Supplement: online supplemental file 1 [file bmjmed-5-1-s001.pdf]

Contents

Figure S1: Association between fragility fracture and time since last bisphosphonate prescription in those prescribed bisphosphonates for three-years. 2

Figure S2: Association between fragility fracture and time since last bisphosphonate prescription in those prescribed bisphosphonates for five-years. 3

Table S1: Association between fragility fractures and oral bisphosphonate prescription interruption by sex 4

Table S2: Association between fragility fractures and bisphosphonate prescription in the 12 months prior to index-date. 5

Table S3: Association between fragility fractures and bisphosphonate prescription considering different thresholds of MPR-I 6

Table S4: Association between fragility fractures and bisphosphonate prescription considering different thresholds of MPR-II 7

Table S5: Association between hip fractures and bisphosphonate prescription interruption 8

Table S6: Association between vertebral fractures and bisphosphonate prescription interruption 9

Table S7: Association between fragility fractures and positive and negative control variables 10

Table S8: Incidence rate of atypical femoral fractures and osteonecrosis of the jaw within the next two-years in patients prescribed bisphosphonates including those with a recorded outcome prior to start of follow-up±. 11

Table S9: Characteristics of study participants stratified by atypical femoral fracture 11

Figure S1: Association between fragility fracture and time since last bisphosphonate prescription in those prescribed bisphosphonates for three-years.

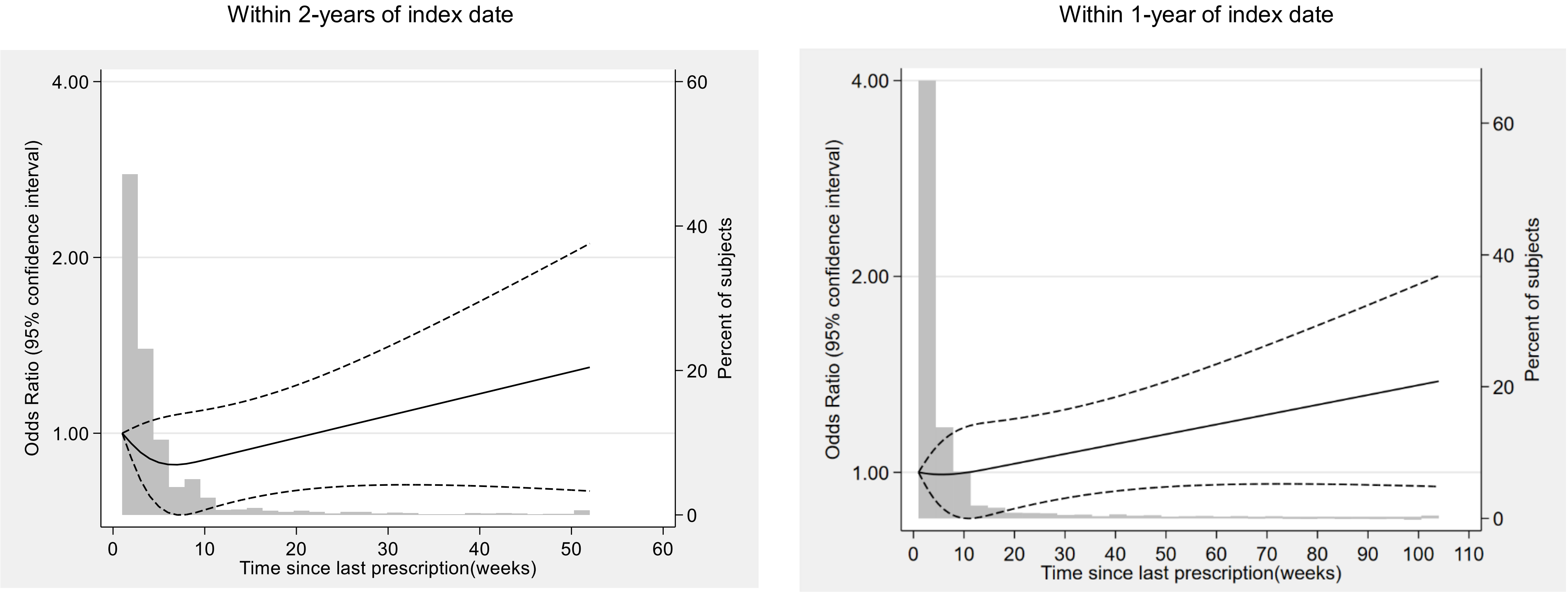

Figure S1 legend: The association between time since last bisphosphonate prescription within 2- years of index date (A) and 1-year of index date (B). The odds ratio of fragility fractures as estimated from an adjusted conditional logistic regression (model 2) with restricted cubic splines in a case-control study nested in the cohort prescribed bisphosphonates for 3-years. The dotted lines represent the 95% confidence interval. The histograms present the distribution of time since last bisphosphonate prescription within 2-years (A) and 1-year of index date (B). Model 2 adjusted for age at start of follow-up, sex, year at start of follow-up, sex, body mass index, ethnicity, region, deprivation, smoking, alcohol intake, autoimmune rheumatic diseases, inflammatory bowel disease, chronic kidney disease stage 3, Charlson comorbidity index, number of primary care consultations and number of hospital admissions in 12 months prior to index date, vitamin D +/-calcium supplement or treatment, and corticosteroids in the previous 90 days of index date.

Figure S2: Association between fragility fracture and time since last bisphosphonate prescription in those prescribed bisphosphonates for five-years.

Within 2-years of index date

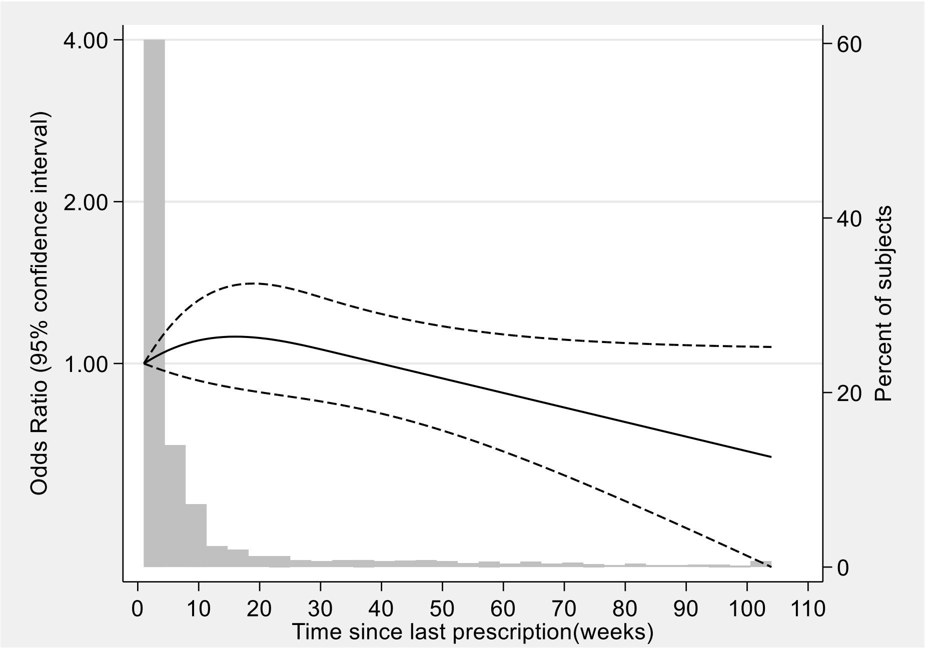

Within 1-year of index date

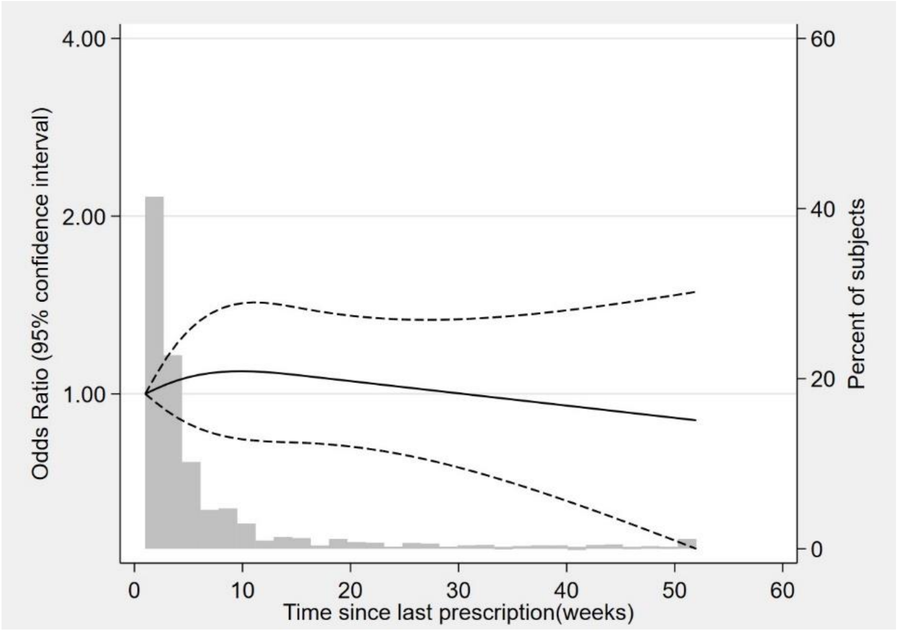

Figure S2 legend: The association between time since last bisphosphonate prescription within 2- years of index date (A) and 1- year of index date (B). The odds ratio of fragility fractures as estimated from an adjusted conditional logistic regression (model 2) with restricted cubic splines in a case-control study nested in the cohort prescribed bisphosphonates for 5-years. The dotted lines represent the 95% confidence interval. The histograms present the distribution of time since last bisphosphonate prescription within two-years (A) and one year of index date (B). Model 2 adjusted for age at start of follow-up, sex, year at start of follow-up, sex, body mass index, ethnicity, region, deprivation, smoking, alcohol intake, autoimmune rheumatic diseases, inflammatory bowel disease, chronic kidney disease stage 3, Charlson comorbidity index, number of primary care consultations and number of hospital admissions in 12 months prior to index date, vitamin D +/-calcium supplement or treatment, and corticosteroids in the previous 90 days of index date.

Table S1: Association between fragility fractures and oral bisphosphonate prescription interruption by sex

	Prior 3-years of bisphosphonate prescription				Prior 5-years of bisphosphonate prescription			
**Exposure window up to 24 months**	**Controls n (%)**	**Cases n (%)**	**Model 1 OR (95% CI)**	**Model 2 OR (95% CI)**	**Controls n (%)**	**Cases n (%)**	**Model 1 OR (95% CI)**	**Model 2 OR (95% CI)**
**Female**								
Continuous prescription (≥ 67%)	4,905 (80.9)	1,188 (77.7)	1	1	2,854 (74.3)	728 (75.1)	1	1
Intermittent prescription (34-66%)	448 (7.4)	143 (9.4)	1.31 (1.08 – 1.60)	1.12 (0.90 – 1.39)	314 (8.2)	75 (7.7)	0.93 (0.71 – 1.21)	0.90 (0.68 – 1.19)
Prescription interruption (0-33%)	713 (11.8)	199 (13.0)	1.19 (0.99 – 1.45)	1.03 (0.84 – 1.26)	671 (17.5)	167 (17.2)	0.98 (0.80 – 1.20)	0.93 (0.75 – 1.16)
**Male**								
Continuous prescription (≥ 67%)	764 (79.4)	194 (78.2)	1	1	432 (75.4)	120 (79.0)	1	1
Intermittent prescription (34-66%)	73 (7.6)	73 (21)	1.13 (0.67 – 1.88)	1.15 (0.64 – 2.06)	49 (8.6)	11 (7.2)	0.79 (0.40 – 1.57)	0.73 (0.33 – 1.65)
Prescription interruption (0-33%)	125 (13.0)	33 (13.3)	1.02 (0.63 – 1.64)	0.93 (0.54 – 1.58)	92 (16.1)	21 (13.8)	0.84 (0.49 – 1.45)	0.81 (0.43 – 1.53)

Model 1 adjusted for matching variables (age at start of follow-up, sex, and year at start of follow-up); Model 2 adjusted for age at start of follow-up, sex, year at start of follow-up, body mass index, ethnicity, region, deprivation, smoking, alcohol intake, autoimmune rheumatic diseases, inflammatory bowel disease, chronic kidney disease stage 3, Charlson comorbidity index, number of primary care consultations in 12 months prior to index date, number of hospital admission in 12 months prior to index date, vitamin D +/-calcium supplement or treatment, and corticosteroids in the previous 90 days of index date.

Table S2: Association between fragility fractures and bisphosphonate prescription in the 12 months prior to index-date.

	Prior 3-years of bisphosphonate prescription				Prior 5-years of bisphosphonate prescription			
**Exposure window up to 24 months**	**Controls n (%)**	**Cases n (%)**	**Model 1 OR (95% CI)**	**Model 2 OR (95% CI)**	**Controls n (%)**	**Cases n (%)**	**Model 1 OR (95% CI)**	**Model 2 OR (95% CI)**
Continuous prescription (≥ 67%)	2,504 (81.7)	606 (76.1)	1	1	1,277 (73.5)	331(74.7)	1	1
Intermittent prescription (34-66%)	212 (6.9)	73 (9.2)	1.39 (1.05-1.85)	1.15 (0.85-1.56)	120 (6.9)	36 (8.1)	1.17 (0.79-1.73)	1.12 (0.74-1.71)
Prescription interruption (0-33%)	348 (11.4)	117 (14.7)	1.22 (0.96-1.56)	1.08 (0.83-1.40)	340 (19.6)	79 (17.2)	0.86 (0.65-1.15)	0.76 (0.55-1.04)

Model 1 adjusted for matching variables (age at start of follow-up, sex, and year at start of follow-up); Model 2 adjusted for age at start of follow-up, sex, year at start of follow-up, body mass index, ethnicity, region, deprivation, smoking, alcohol intake, autoimmune rheumatic diseases, inflammatory bowel disease, chronic kidney disease stage 3, Charlson comorbidity index, number of primary care consultations in 12 months prior to index date, number of hospital admission in 12 months prior to index date, vitamin D +/-calcium supplement or treatment, and corticosteroids in the previous 90 days of index date.

Table S3: Association between fragility fractures and bisphosphonate prescription considering different thresholds of MPR-I

	Prior 3-years of bisphosphonate prescription				Prior 5-years of bisphosphonate prescription			
**Exposure window up to 24 months**	**Controls n (%)**	**Cases n (%)**	**Model 1 OR (95% CI)**	**Model 2 OR (95% CI)**	**Controls n (%)**	**Cases n (%)**	**Model 1 OR (95% CI)**	**Model 2 OR (95% CI)**
Continuous prescription with optimal adherence (≥ 80%)	5,198 (74.0)	1,242 (70.0)	1	1	3,028 (68.6)	775 (69.1)	1	1
Continuous prescription with acceptable adherence (67-79%)	471 (6.7)	140 (7.9)	1.25 (1.02,1.54)	1.11 (0.90,1.38)	258 (5.9)	73 (6.5)	1.11 (0.84,1.47)	1.10 (0.82,1.47)
Intermittent prescription (34-66%)	521 (7.4)	164 (9.2)	1.31 (1.09,1.58)	1.14 (0.93,1.39)	363 (8.2)	86 (7.7)	0.92 (0.72,1.18)	0.87 (0.67,1.14)
Prescription interruption (0-33%)	838 (11.9)	232 (13.1)	1.19 (1.00,1.42)	1.04 (0.86,1.25)	763 (17.3)	188 (16.8)	0.97 (0.80,1.18)	0.93 (0.75,1.14)

Model 1 adjusted for matching variables (age at start of follow-up, sex, and year at start of follow-up); Model 2 adjusted for age at start of follow-up, sex, year at start of follow-up, body mass index, ethnicity, region, deprivation, smoking, alcohol intake, autoimmune rheumatic diseases, inflammatory bowel disease, chronic kidney disease stage 3, Charlson comorbidity index, number of primary care consultations in 12 months prior to index date, number of hospital admission in 12 months prior to index date, vitamin D +/-calcium supplement or treatment, and corticosteroids in the previous 90 days of index date.

Table S4: Association between fragility fractures and bisphosphonate prescription considering different thresholds of MPR-II

	Prior 3-years of bisphosphonate prescription				Prior 5-years of bisphosphonate prescription			
**Exposure window up to 24 months**	**Controls n (%)**	**Cases n (%)**	**Model 1 OR (95% CI)**	**Model 2 OR (95% CI)**	**Controls n (%)**	**Cases n (%)**	**Model 1 OR (95% CI)**	**Model 2 OR (95% CI)**
Continuous prescription with high adherence (≥ 75%)	5,414 (77.0)	1,313 (73.9)	1	1	3,147 (71.3)	801 (71.4)	1	1
Continuous prescription with medium adherence (50-74%)	558 (7.9)	165 (9.3)	1.22 (1.01,1.47)	1.05 (0.86,1.28)	343 (7.8)	100 (8.9)	1.14 (0.90,1.45)	1.13 (0.88,1.46)
Intermittent prescription (25-49%)	289 (4.1)	87 (4.9)	1.24 (0.96,1.59)	1.09 (0.84,1.42)	220 (5.0)	49 (4.4)	0.88 (0.64,1.21)	0.80 (0.57,1.13)
Prescription interruption (0-24%)	767 (10.9)	213 (12.0)	1.18 (0.98,1.43)	1.04 (0.85,1.27)	702 (15.9)	172 (15.3)	0.97 (0.79,1.18)	0.92 (0.74,1.14)

Model 1 adjusted for matching variables (age at start of follow-up, sex, and year at start of follow-up); Model 2 adjusted for age at start of follow-up, sex, year at start of follow-up, body mass index, ethnicity, region, deprivation, smoking, alcohol intake, autoimmune rheumatic diseases, inflammatory bowel disease, chronic kidney disease stage 3, Charlson comorbidity index, number of primary care consultations in 12 months prior to index date, number of hospital admission in 12 months prior to index date, vitamin D +/-calcium supplement or treatment, and corticosteroids in the previous 90 days of index date.

Table S5: Association between hip fractures and bisphosphonate prescription interruption

	Prior 3-years of bisphosphonate prescription				Prior 5-years of bisphosphonate prescription			
**Exposure window up to 24 months**	**Controls n (%)**	**Cases n (%)**	**Model 1 OR (95% CI)**	**Model 2 OR (95% CI)**	**Controls n (%)**	**Cases n (%)**	**Model 1 OR (95% CI)**	**Model 2 OR (95% CI)**
**Overall**								
Continuous prescription (≥ 67%)	1,425 (79.2)	330 (72.2)	1	1	725 (79.0)	182 (77.8)	1	1
Intermittent prescription (34-66%)	127 (7.1)	54 (11.8)	1.81 (1.29,2.54)	1.47 (1.00,2.16)	58 (6.3)	17 (7.3)	1.15 (0.65,2.04)	1.06 (0.56,2.02)
Prescription interruption (0-33%)	247 (13.7)	73 (16.0)	1.33 (0.96,1.84)	1.06 (0.73,1.53)	135 (14.7)	35 (15.0)	1.03 (0.66,1.59)	0.85 (0.51,1.40)
**Female**								
Continuous prescription (≥ 67%)	1,224 (79.4)	281 (72.2)	1	1	623 (78.5)	157 (78.1)	1	1
Intermittent prescription (34-66%)	113 (7.4)	46 (11.8)	1.74 (1.21,2.52)	1.42 (0.94,2.17)	52 (6.6)	13 (6.5)	1.00 (0.53,1.90)	1.10 (0.54,2.25)
Prescription interruption (0-33%)	198 (12.9)	62 (15.9)	1.46 (1.02,2.07)	1.12 (0.75,1.68)	119 (15.0)	31 (15.4)	1.02 (0.64,1.63)	0.89 (0.52,1.53)
**Male**								
Continuous prescription (≥ 67%)	201 (76.1)	49 (72.1)	1	1	102 (82.3)	25 (75.8)	1	1
Intermittent prescription (34-66%)	14 (5.3)	8 (11.8)	2.36 (0.93,5.97)	1.43 (0.39,5.22)	6 (4.8)	-/- (12.1)	2.28 (0.58,8.9)	-
Prescription interruption (0-33%)	49 (18.6)	11 (16.2)	0.84 (0.37,1.92)	0.99 (0.322,3.05)	16 (12.9)	-/- (12.1)	1.06 (0.31,3.67)	-

Model 1 adjusted for matching variables (age at start of follow-up, sex, and year at start of follow-up); Model 2 adjusted for age at start of follow-up, sex, year at start of follow-up, body mass index, ethnicity, region, deprivation, smoking, alcohol intake, autoimmune rheumatic diseases, inflammatory bowel disease, chronic kidney disease stage 3, Charlson comorbidity index, number of primary care consultations in 12 months prior to index date, number of hospital admission in 12 months prior to index date, vitamin D +/-calcium supplement or treatment, corticosteroids in the previous 90 days of index date. ; -/- value < 5; - not possible to execute a fully adjusted model due to few cases.

Table S6: Association between vertebral fractures and bisphosphonate prescription interruption

	Prior 3-years of bisphosphonate prescription				Prior 5-years of bisphosphonate prescription			
**Exposure window**	**Controls n (%)**	**Cases n (%)**	**Model 1 OR (95% CI)**	**Model 2 OR (95% CI)**	**Controls n (%)**	**Cases n (%)**	**Model 1 OR (95% CI)**	**Model 2 OR (95% CI)**
Continuous prescription (≥ 67%)	1,393 (81.8)	355 (82.8)	1	1	897 (72.4)	236 (74.9)	1	1
Intermittent prescription (34-66%)	124 (7.3)	34 (7.9)	1.08 (0.72,1.60)	0.92 (0.60,1.41)	121 (9.8)	23 (7.3)	0.72 (0.45,1.15)	0.69 (0.42,1.14)
Prescription interruption (0-33%)	186 (10.9)	40 (9.3)	0.80 (0.53,1.22)	0.63 (0.41,0.99)	221 (17.8)	56 (17.8)	0.98 (0.68,1.39)	0.81 (0.55,1.19)

Model 1 adjusted for matching variables (age at start of follow-up, sex, and year at start of follow-up); Model 2 adjusted for age at start of follow-up, sex, year at start of follow-up, body mass index, ethnicity, region, deprivation, smoking, alcohol intake, autoimmune rheumatic diseases, inflammatory bowel disease, chronic kidney disease stage 3, Charlson comorbidity index, number of primary care consultations in 12 months prior to index date, number of hospital admission in 12 months prior to index date, vitamin D +/-calcium supplement or treatment, and corticosteroids in the previous 90 days of index date.

Table S7: Association between fragility fractures and positive and negative control variables

	Prior 3-years of bisphosphonate prescription				Prior 5-years of bisphosphonate prescription			
**Exposure window up to 24 months**	**Controls n (%)**	**Cases n (%)**	**Model 1 OR (95% CI)**	**Model 2 OR (95% CI)**	**Controls n (%)**	**Cases n (%)**	**Model 1 OR (95% CI)**	**Model 2 OR (95% CI)**
**Opioid prescription (Positive control variable)**								
Remote or no prescription	4,672 (66.5)	934 (52.5)	1	1	2,962 (67.1)	596 (53.1)	1	1
Within 91 to 365.25 days	832 (11.8)	201 (11.3)	1.22 (1.03,1.45)	1.04 (0.87, 1.25)	498 (11.3)	130 (11.6)	1.32 (1.06, 1.63)	1.09 (0.87, 1.37)
Within 90 days	1,524 (21.7)	643 (36.2)	2.13 (1.89,2.40)	1.87 (1.65, 2.13)	952 (21.6)	396 (35.3)	2.12 (1.83, 2.46)	1.82 (1.55, 2.14)
**Osteoarthritis (Negative control variable)**								
No	4,375 (62.3)	1,062 (59.7)	1	1	2,652 (60.1)	655 (58.4)	1	1
Yes	2,653 (37.8)	716 (40.3)	1.12 (1.01, 1.25)	1.05 (0.94, 1.18)	1,760 (39.9)	467 (41.6)	1.08 (0.94, 1.23)	1.07 (0.92, 1.24)

Model 1 adjusted for matching variables (age at start of follow-up, sex, and year at start of follow-up); Model 2 adjusted for age at start of follow-up, sex, year at start of follow-up, body mass index, ethnicity, region, deprivation, smoking, alcohol intake, autoimmune rheumatic diseases, inflammatory bowel disease, chronic kidney disease stage 3, Charlson comorbidity index, number of primary care consultations in 12 months prior to index date, number of hospital admission in 12 months prior to index date, vitamin D +/-calcium supplement or treatment, and corticosteroids in the previous 90 days of index date

Table S8: Incidence rate of atypical femoral fractures and osteonecrosis of the jaw within the next two-years in patients prescribed bisphosphonates including those with a recorded outcome prior to start of follow-up±.

	Number of outcomes	Person-time (years)	Incidence rate (95% CI/1000 person-years
**Atypical femoral fractures**			
3-year prescription cohort	85	40,924	2.08 (1.68-2.57)
5-year prescription cohort	59	22,227	2.65 (2.06-3.43)
**Osteonecrosis of the jaw**			
3-year prescription cohort	6	125,517	0.05 (0.02-0.11)
5-year prescription cohort	-/-	98,135	-/-

±Start of follow-up is three or five years after the first bisphosphonate prescription in the three- or five-year cohort respectively.

-/-Number of observations less than 5 are suppressed as per CPRD requirements to maintain patient anonymity.

Table S9: Characteristics of study participants stratified by atypical femoral fracture

	3-year prescription cohort	5-year prescription cohort

	(n=26,839)		(n=14,745)	
	No<br>n (%)	Yes<br>n (%)	No<br>n (%)	Yes<br>n (%)
Sex				
Male	5,076 (19.0)	12 (14.1)	2,568 (17.5)	9 (15.3)
Female	21,678 (81.0)	73 (85.9)	12,118 (82.5)	50 (84.8)
Age (years)				
<65	4,774 (17.8)	8 (9.4)	2,169 (14.8)	-/- (6.8)
65-79	11,750 (43.9)	31 (36.5)	6,629 (45.1)	23 (39.0)
≥80	10,230 (38.2)	46 (54.1)	5,888 (40.1)	32 (54.2)
Body mass index (kg/m^2)				
<18.5	1,105 (4.1)	9 (10.6)	621 (4.2)	-/- (1.7)
18.5-24.9	11,134 (41.6)	26 (30.6)	6,359 (43.3)	24 (40.7)
25.0-29.9	8,284 (30.9)	26 (30.6)	4,596 (31.3)	20 (33.9)
≥30	4,477 (16.7)	14 (16.5)	2,413 (16.4)	10 (17.0)
Missing data	1,754 (6.6)	10 (11.8)	697 (4.8)	-/- (6.8)
Smoking status				
Non-smoker	16,342 (61.1)	54 (63.5)	9,166 (62.4)	38 (64.4)
Current smoker	2,322 (8.7)	9 (10.6)	1,142 (7.8)	6 (10.2)
Ex-smoker	7,862 (29.4)	20 (23.5)	4,333 (29.5)	14 (23.7)
Missing data	228 (0.9)	-/- (2.4)	45 (0.3)	-/- (1.7)
Alcohol consumption (units per week)				
None	5,092 (19.0)	15 (17.7)	2,907 (19.8)	16 (27.1)
Low (1-14)	11,230 (42.0)	25 (29.4)	6,119 (41.7)	23 (39.0)
Moderate (15-21	879 (3.3)	-/- (3.5)	490 (3.3)	-/- (0.0)
Hazardous (>21)	1,381 (5.2)	-/- (2.4)	748 (5.1)	-/- (3.4)
Former drinker	3,017 (11.3)	10 (11.8)	1,802 (12.3)	7 (11.9)
Missing data	5,155 (19.3)	30 (35.3)	2,620 (17.8)	11 (18.6)
Ethnicity				
Non-white	2,659 (9.9)	-/- (1.2)	1,499 (10.2)	-/- (5.1)
White	24,095 (90.1)	84 (98.8)	13,187 (89.8)	56 (94.9)
Bisphosphonate				
Alendronate	23,146 (86.5)	67 (78.8)	12,627 (86.0)	49 (83.1)

Risedronate	3,608 (13.5)	18 (21.2)	2,059 (14.0)	10 (17.0)
Previous fragility fracture				
No	15,122 (56.5)	30 (35.3)	8,284 (56.4)	21 (35.6)
Yes	11,632 (43.5)	55 (64.7)	6,402 (43.6)	38 (64.4)
Number of corticosteroid prescriptions≠				
0	20,287 (75.8)	71 (83.5)	11,604 (79.0)	45 (76.3)
1-5	2,654 (9.9)	6 (7.1)	1,324 (9.0)	8 (13.6)
≥6	3,813 (14.3)	8 (9.4)	1,758 (12.0)	6 (10.2)

-/-Number of observations less than 5 are suppressed as per CPRD requirements to maintain patient anonymity; ≠Number of corticosteroid prescriptions in the 12 months prior to start of the 2-years of follow-up.
