# Supplementary material for: Fragility fracture, atypical femoral fracture, and osteonecrosis of jaw after bisphosphonate prescription for three and five years, based on primary and secondary care data in England: nested case-control and cohort studies
Source: BMJ Med. 2026 Apr 22;5(1):e002085. doi: 10.1136/bmjmed-2025-002085 (PMC13110635; doi:10.1136/bmjmed-2025-002085)
Supplement: Supplementary file 3 [file bmjmed-2025-002085.reviewer_comments.pdf]

PEER REVIEW HISTORY

BMJ Medicine publishes all reviews undertaken for accepted manuscripts. Reviewers are asked to complete a checklist review form and are provided with free text boxes to elaborate on their assessment. These free text comments are reproduced below.

ARTICLE DETAILS

**TITLE (PROVISIONAL)**	Fragility fracture, atypical femoral fracture, and osteonecrosis of jaw after bisphosphonate prescription for three- and five-years: nested case-control and cohort studies using linked English primary-care, hospitalisation and mortality data.
**AUTHORS**	Kukreja, Niraj; Nakafero, Georgina; McKeever, Tricia; Welsh, Victoria; Muller, Sara; Abhishek, Abhishek

VERSION 1 - REVIEW

**REVIEWER NAME**	Singh, Ruhani
**REVIEWER AFFILIATION**	National Medical Research Association
**REVIEWER CONFLICT OF INTEREST**	None
**AI DISCLOSURE**	No
**DATE REVIEW RETURNED**	26-Sep-2025

**GENERAL COMMENTS**	This is an important clinical question with clear policy relevance. The study leverages high-quality linked data, prespecifies a rich set of covariates and multiple sensitivity analyses, and uses a transparent ascertainment algorithm for ONJ. Overall, the work is promising and potentially practice-informing, but several methodological and reporting issues should be addressed to strengthen interpretation and clinical applicability.<br><br>Comments:<br>1. The definitions for MPR are arbitrary and would benefit from further justification as to why they were chosen (since the percentages used do not match the source cited). Alternative MPR definitions or continuous MPR splices can be explored to test this interaction. Furthermore, since requirement of >67% MPR will lead to selection of healthier patients who are consistently adherent to therapy, it may exclude patients who are discontinuing earlier and thus have differential fracture risk. While this has been covered to some extent in Supplementary material, further clarification would help to clarify which patients face fracture risk.<br><br>2. Ascertainment of ONJ using algorithm from Persson et al is appropriate, but given the rarity of ONJ ascertained, it may be helpful to explore the breakdown of strong vs weak codes to identify confirmatory codes. Given the rarity of ONJ, the authors should also explicitly discuss the imprecision around incidence estimates<br><br>3. This study would benefit from a positive and negative control variable to identify differences and probe for residual confounding.<br><br>4. If ethnicity is available, examine interaction with sex and deprivation to determine equity in associations.

	5. Check spelling and language carefully - for example the FDA is incorrectly labelled Food and Drug Agency when it should be Administration.

**REVIEWER NAME**	Kim, Jin-Woo
**REVIEWER AFFILIATION**	Ewha Womans University
**REVIEWER CONFLICT OF INTEREST**	None
**AI DISCLOSURE**	Yes - For English proof-reading
**DATE REVIEW RETURNED**	27-Sep-2025

**GENERAL COMMENTS**	This nested case–control and cohort analysis in CPRD Aurum evaluated whether interrupting oral bisphosphonates after 3 or 5 years of adherent use increases fragility fracture risk over the next two years, and estimated the incidence of atypical femoral fracture (AFF) and osteonecrosis of the jaw (ONJ). Using MPR-based exposure categories (≥67% "continued," 34–66% "intermittent," ≤33% "interruption"), the authors report no association between treatment interruption and fragility fracture (e.g., prior-3y cohort: aOR 1.02 [0.85–1.24] vs continued) and very low two-year ONJ rates (0.07/1,000 person-years after 3y; none observed after 5y), with AFF incidence ~2 per 1,000 person-years.<br><br>1. Define "holiday" more precisely (time since last dose). Your exposure is MPR buckets (≤33%, 34–66%, ≥67%), which reflects prescribing, not pharmacologic off time. Please re express exposure as time since last bisphosphonate prescription (TSL), modelled continuously (restricted cubic splines), alongside your MPR groups. This addresses dilution from short "interruptions." (Refer to https://www.nature.com/articles/s41467-025-59718-x)<br><br>2. Target trial emulation to reduce healthy adherer/landmark bias. Recasting the design as a clone censor weight comparison of policies ("continue" vs "pause ≤2y") with stabilized IPTW would better handle selection on surviving/adherent users (MPR≥67% for 3/5y). Report absolute risks at 1 and 2years.<br><br>3. Regarding ONJ ascertainment, validate and stratify by procedure. Your two step "strong/weak jaw codes" algorithm is sensible; please show PPV using a small validation sample and stratify ONJ by recent tooth extraction (if OPCS/primary care procedure codes are available), where risk clusters.<br><br>4. ONJ precision & rare event methods. ONJ events are extremely few within 2years (0.07/1,000py after 3y; none after 5y). Use penalized likelihood (Firth) and present wider windows (5–10y) in main text (currently in tables), plus exact counts where disclosure rules allow.<br><br>5. Integrate benefit–harm framing. Place absolute fracture risks (no increase with interruption) alongside absolute ONJ/AFF rates (e.g., AFF 1.93 and 2.50/1,000py; ONJ ≈0–0.07/1,000py over 0–2y) with numbers needed to treat/harm for "continue vs pause."<br><br>6. Agent specific heterogeneity. You note more ONJ on alendronate and higher AFF on risedronate in some strata; provide fully adjusted agent specific effects for "continue vs pause," and interaction P values.

	7. Confounding by unmeasured bone status. Without BMD or vertebral morphometry, risk stratification is limited. Consider proxies (prior vertebral fracture codes, height loss, high dose steroids), perform E values, and a negative control outcome analysis (e.g., non skeletal events).<br><br>8. Outcome definition hierarchy. Report results when restricting fragility fractures to hip only (your sensitivity suggests similar null for interruption; intermittent shows borderline higher hip risk) and make this a co primary or key secondary analysis.<br><br>9. Competing risks & mortality. In this elderly cohort, use cause specific or subdistribution hazard models for fracture and ONJ across 5–10y horizons to account for death as a competing event.<br><br>10. Transparency of code lists. Move the long code lists (TableS1) into a public repository with versioning and upload the analytic code used for ONJ adjudication.

**REVIEWER NAME**	Zhang, Xiaowen
**REVIEWER AFFILIATION**	University of Hong Kong Faculty of Medicine, Department of Pharmacology and Pharmacy
**REVIEWER CONFLICT OF INTEREST**	None
**AI DISCLOSURE**	No
**DATE REVIEW RETURNED**	28-Sep-2025

**GENERAL COMMENTS**	Nakafero et al. assessed the fragile fracture risk after bisphosphonate discontinuation at 3 and 5 years and the incidence ONJ and AFF the rare adverse effects associated with bisphosphanate use. The study was based on the large population-based EHR CDRP in the UK and used nested case-control design to address a clinically relevant question in the osteoporosis management.<br><br>Major limitations:<br>1. The reliance on MPR ≥67% is unconventional in osteoporosis pharmacoepidemiology. MPR could be inflated in some cases.<br>2. The 2-year averaging MPR approach obscures some true discontinuation (e.g., a 95-day gap could be missed using MPR). Using the more standard Proportion of Days Covered (PDC) and defining discontinuation via a persistence rule with a pre-specified grace period might be more accurate.<br>3. Handling switches to other anti-osteoporosis medications (e.g., denosumab, teriparatide, HRT) was not specified in the methods.<br>4. The nested case-control design offers a powerful opportunity to investigate a dose-response relationship based on the duration of the treatment gap and the timing of the gap, but they were not done in the current study. Exploring these would add novelty to the findings.<br>5. Important covariates such as BMD, prior fracture severity, physical activity, fall-related risk factors e.g. use of drugs (PPIs, antipsychotics) were not adjusted in the analyses. Although some may be restricted by the data availalbility (BMD was discussed in the study limitation), it may restrain the intepretation of the results and clinical impact.<br>6. Outcomes: the authors did hip fracture individually. How about

	other sites e.g. vertebral fractures?<br>7. A subgroup analysis by sex would be highly valuable, given the predominant use of bisphosphonates in women.<br>8. Broaderline OR of hip fracture risk (1.47, 1.00–2.16) and warrants more detailed discussion, and suggest do a female subgroup analysis.<br>9. The statement implying “no increase in fragility fractures within two years after discontinuation” may be somewhat strong given the exposure definition, residual confoundings, and borderline hip fracture result. It may be interpreted as a two-year drug gap but the study used MPR.<br><br>Minor comments:<br>1. Correct typos (e.g., Table 2 title) and ensure the abstract’s ORs for 3-year and 5-year analyses match the main tables.<br>2. In Figure 2, the exclusion criteria list "denosumab," which is not a bisphosphonate.<br>3. Providing a statistical comparison of ONJ and AFF risk between the discontinuation and continuation groups would be more interesting.

**REVIEWER NAME**	Persson, Rebecca
**REVIEWER AFFILIATION**	Boston Collaborat Drug Surveillance Program
**REVIEWER CONFLICT OF INTEREST**	None
**AI DISCLOSURE**	No
**DATE REVIEW RETURNED**	10-Oct-2025

**GENERAL COMMENTS**	This study in the CPRD assessed the effect of bisphosphonate treatment holidays on fracture risk as well as AFF and ONJ using case-control and cohort analyses. These analyses appear to be well conducted and this study will add useful information for patients and clinicians. I do have a few comments for the authors’ consideration.<br><br>Major comments:<br>In the limitations section lines 38-50 the authors write about the possibility of misclassification of exposure due to specialist treatments (which are not captured in the CPRD), in particular, the potential of zoledronic acid or denosumab exposed patients to be misclassified as treatment interrupted patients. This is a concern that requires more consideration than the authors have provided as this misclassification, if extreme, could entirely explain the null observed effect for fracture risk. The authors should consider quantitative bias analysis or other method to assess whether or not their observed results could be a result of exposure misclassification. For example: How many patients from the treatment interruption group would have to have been misclassified for a true effect to be erased? Is there any reason to think misclassification could be different for cases and controls? etc.<br><br>The authors use the term adherence throughout the paper. It is important for the authors to state at some point in the manuscript that CPRD contains records of prescriptions written, not prescriptions dispensed, and not pills taken and thus that adherence levels have been assumed based on repeated prescriptions.<br><br>Discussion section: “Almost all the AFF and ONJ outcomes were ascertained in hospitalization and mortality records. The positive

	predictive value of AFF recorded in hospital discharge summaries is reported to range between 90%-92% (27, 28). This increases the validity of our findings." This is somewhat misleading as references 27 and 28 are validation studies of codes for "Subtrochanteric femur or femoral shaft fracture" and "Subtrochanteric or Diaphyseal Femoral Fractures", respectively, not AFF specifically. The authors should clarify that all of these cases are assumed to be atypical due to bisphosphonate exposure. It would strengthen their AFF case definition if they excluded any cases with major accidents occurring around the time of the fracture event. However, it is reassuring that the inclusion of any typical femoral fractures as AFF would have overestimated the rates of AFF and thus would not change the overall conclusion that AFF was an uncommon adverse event.<br><br>Results for Incidence of AFF, lines 40-45: the statement that the incidence of AFF as higher with prescription steroids is not consistent with the data presented in Table 3. Please revise.<br><br>Minor comments:<br>Introduction: 2nd sentence is very long and hard to parse. Consider revision.<br>It is unclear if the >5 year cohort includes patients in the >3 year cohort. Please describe any overlap.<br>Figure 1: Please clarify index date and exposure assessment period<br>Table 2: Right column header should be "Prior 5-years", correct?<br>Table 3: Clarify with words or improved formatting which analyses are for the 2-year ascertainment period and which are for other ascertainment periods.

**REVIEWER NAME**	Perera, Rafael
**REVIEWER AFFILIATION**	University of Oxford, Primary Care Health Sciences
**REVIEWER CONFLICT OF INTEREST**	None
**AI DISCLOSURE**	No
**DATE REVIEW RETURNED**	30-Jan-2026

**GENERAL COMMENTS**	Stats Review:<br><br>The current manuscript presents results from a cohort study based on CPRD (UK primary care) data and two nested case-control studies (varying treatment duration) to estimate the prevalence of fragility fractures (and similar outcomes) and evaluate the impact that stopping (or reducing) bisphosphonate treatment has on the risk of sustaining these types of fractures. The authors find that in this population, the risk of these outcomes do not increase or decrease after treatment discontinuation (or reduction). The findings appear mostly in line with previous research and appear credible. There are some clarifications required in the form the studies are reported which should help put the clinical implications in the right perspective.<br><br>Issues:<br>- The definition of the cohort is based around a medication possession ratio (MPR) > 2/3 of the time (67%). This measure is a proxy for adherence and is treated as a categorical variable (with some extra sensitivity analyses that modify the threshold at which these categories are defined). The use of categories is likely to reduce the power of the measure to detect different risk levels and

	there needs to be a justification for this approach. At the same time, perhaps surprisingly, the 'middle category' appears to present an increased risk (but not at the extremes). Some discussion about the reason for this observed difference is required. Regarding this point, please comment about the potential effect that might exist and would not be detectable given the data available (as the confidence intervals for the Odds Ratios are not very precise.<br>- From the description, continuous MPR > 67% appears to be required to enter the nested case-control studies (3 and 5-years). Please clarify what approach is used during the follow up (2 year) period. In particular if different MPR category levels are registered during this period, how was this incorporated into the analysis?<br>- The two nested case-control studies as defined appear to suggest that the 5-year treatment cohort is a subset of the 3-year one. Please clarify if this is correct and comment on what implications this has in relation to the lack of independence of the data and how this should be interpreted.<br>- Please also provide a justification for the use of a 2-year follow-up.<br>- The fragility fracture outcome appears to potentially occur at any point during the treatment (cohort definition) or follow-up intervals. Please explain if this is the case and also what implications this has for the analysis? Previous fragility fracture does not appear to be one of the covariates included in the model but it would be expected that this would be an important risk modifier for future fragility fractures. Please clarify and justify the approach taken.<br>- The statement 'Our findings suggest that in those with prior fragility fractures,<br>treatment interruption for up to two years could be considered after a minimum of three years of treatment.', seems to me to be an overinterpretation of the data. Please provide justification.<br>- For Table S5, the percentage given for BMI category 25-29.9 '(41%)' is incorrect. Please check data in table.

VERSION 1 – AUTHOR RESPONSE

Reviewer: 1

Dr. Ruhani Singh, National Medical Research Association

Comments to the Author

This is an important clinical question with clear policy relevance. The study leverages high-quality linked data, prespecifies a rich set of covariates and multiple sensitivity analyses, and uses a transparent ascertainment algorithm for ONJ. Overall, the work is promising and potentially practice-informing, but several methodological and reporting issues should be addressed to strengthen interpretation and clinical applicability.

Response: Thank you for the supportive comments.

Comments:

1. (a) The definitions for MPR are arbitrary and would benefit from further justification as to why they were chosen (since the percentages used do not match the source cited). Alternative MPR definitions or continuous MPR splices can be explored to test this interaction.

Response: Thank you for asking us to clarify.

We have added the below in methods:

We chose a slightly higher MPR (≥67%) to define continued prescription than the 50% refill compliance (i.e., collection of medication) that was reported to associate with fewer fragility fractures. This is because we derived MPR from prescription data, regardless of whether the medicine was collected or not. Unfortunately, data on whether a prescribed medicine is collected or not is not available in the CPRD. We anticipated a small proportion of patients to not collect their prescriptions and therefore chose a slightly higher MPR cut-off.

We have added the below in the section on sensitivity analysis:

Third, we used different thresholds of MPR i.e., 0-24%, 25-49%, 50-74%, and ≥75%.

We have added the below in results:

Findings were similar when continued prescription was defined using MPR ≥80% within two years, when different levels of MPR were considered to define exposure status, and on considering bisphosphonate prescription interruption within the 12-month period prior to index date (Table S2-S4).

1. (b) Furthermore, since requirement of >67% MPR will lead to selection of healthier patients who are consistently adherent to therapy, it may exclude patients who are discontinuing earlier and thus have differential fracture risk. While this has been covered to some extent in Supplementary material, further clarification would help to clarify which patients face fracture risk.

Response: We agree, the results of this study should not apply to people with lower levels of MPR. We have added a discussion point.

Patients included in the nested case control studies had high MPR in the run-in phase. These findings should not be extrapolated to those with lower levels of medication possession as they may still be at increased risk of fragility fractures.

2. Ascertainment of ONJ using algorithm from Persson et al is appropriate, but given the rarity of ONJ ascertained, it may be helpful to explore the breakdown of strong vs codes to identify confirmatory codes. Given the rarity of ONJ, the authors should also explicitly discuss the imprecision around incidence estimates.

Response: Thank you for the opportunity to clarify. We have added the below in results. Please note the numbers in parentheses have been suppressed from the manuscript given CPRD disclosure rules when there are fewer than 5 events:

Most ONJ cases ascertained in this study (78.3% (18 of 23) in three-year cohort and 77.8% (14 of 18) in five-year cohort) had strong codes as defined by Persson *et al*.

and in discussion:

There were few ONJ outcomes ascertained resulting in imprecise estimates and the incidence of ONJ reported by us should be interpreted with caution.

3. This study would benefit from a positive and negative control variable to identify differences and probe for residual confounding.

Response: Thank you for asking us to do so. We have undertaken the suggested analysis.

Please see methods:

Positive and negative control exposure: Opioid prescription (categorised as recent (≥1 prescription within 90 days of index date), remote (≥1 prescription within 91-365 days of index date), and either past or no prescription) was a positive control exposure as it is associated with fragility fracture (17). A prior diagnosis of osteoarthritis (yes, no) was a negative control exposure as this is not associated with fragility fractures (18).

Please see results:

A prescription of opioids within the previous 90 days was associated with an increased risk of fragility fracture while a diagnosis of osteoarthritis was not associated with fragility fracture (Table S7).

4. If ethnicity is available, examine interaction with sex and deprivation to determine equity in associations.

Response: We did not find significant interaction between exposure and deprivation. This was stated in the results section. As suggested by the reviewer we have undertaken further analysis to explore the interaction between exposure and (a) sex, and (b) ethnicity. Approximately 93-96% cases and controls were White and there was a small number of people from other ethnicities. This only allows us to explore ethnicity as a two-level variable (White/non-white).

Please see methods:

We tested for interactions between pre-specified prognostic factors i.e., age (≤70 vs >70 years), sex (female or male), deprivation (first two vs last three quintiles), drug (alendronate or risedronate), ethnicity (White or non-white), prior fragility fracture, prior hip fracture, and bisphosphonate prescription status up to two years before the index-date in the fully adjusted model. Approximately 95% of study population was White and there were few people from many other ethnicities. This allowed us to explore ethnicity as a two-level variable (White/non-white).

Please see results:

Interaction tests for age, sex, deprivation, ethnicity, drug, prior fragility fractures, and prior hip fractures were not significant with P-value 0.788, 0.942, 0.827, 0.301, 0.852, 0.484, 0.167 respectively in the cohort prescribed bisphosphonates for three years and with p-value 0.216, 0.861, 0.896, 0.631, 0.559, 0.853 and 0.632 respectively, in the cohort prescribed bisphosphonates for five years.

5. Check spelling and language carefully - for example the FDA is incorrectly labelled Food and Drug Agency when it should be Administration.
Response: Apologies, this has been corrected.

Reviewer: 2
Dr. Jin-Woo Kim, Ewha Womans University
Comments to the Author

1. Define “holiday” more precisely (time since last dose). Your exposure is MPR buckets (≤33%, 34–66%, ≥67%), which reflects prescribing, not pharmacologic off time. Please re express exposure as time since last bisphosphonate prescription (TSL), modelled continuously (restricted cubic splines), alongside your MPR groups. This addresses dilution from short “interruptions.” (Refer to https://www.nature.com/articles/s41467-025-59718-x)

Response: We chose categories of MPR, as we set out to compare fragility fracture risk in those prescribed or not prescribed bisphosphonates. This is clinically more meaningful than MPR as a continuous variable. We also looked at cumulative bisphosphonate prescription.

We previously analysed time since last prescription as a continuous variable in a linear model (Please see Table 2). Based on your comment we modelled this relationship using restricted cubic splines and found evidence for non-linear association. We now report these results and have removed results of linear model.

See section on statistical analysis:

Multivariable conditional logistic regression was used to assess the association between fragility fracture and bisphosphonate prescription interruption and cumulative bisphosphonate prescription. Association with time since last bisphosphonate prescription was estimated from a conditional logistic regression model with restricted cubic splines.

See section on results:

Increasing time since last bisphosphonate prescription was not associated with fragility fracture in the cohort prescribed bisphosphonates for three-years (Figure S1) or five-years (Figure S2).

2. Target trial emulation to reduce healthy adherer/landmark bias. Recasting the design as a clone censor weight comparison of policies (“continue” vs “pause ≤2y”) with stabilized IPTW would better handle selection on surviving/adherent users (MPR≥67% for 3/5y). Report absolute risks at 1 and 2years.

Response: Thank you for raising this important design consideration. We considered target trial emulation at length but decided not to proceed with this because

(a) patients could stop and restart treatment on more than one occasion making cloning and censoring difficult to implement and interpret given the long retention of bisphosphonates as in patients prescribed bisphosphonates for 3-5 years, there is prolonged drug retention and pharmacologic effect for 1-2 years even after treatment is stopped,
(b) mandating an event free 2-year survival in both arms to study comparison of policies as suggested would result in a study population that is quite unusual in that they will be at low-risk of fragility fractures and the results would not be generalisable, and
(c) mandating a 2-year event free follow-up in the pause group alone would result in immortal time bias.

We hope this rationale is sufficient to address the reviewer concerns.

3. Regarding ONJ ascertainment, validate and stratify by procedure. Your two step “strong/weak jaw codes” algorithm is sensible; please show PPV using a small validation sample and stratify ONJ by recent tooth extraction (if OPCS/primary care procedure codes are available), where risk clusters.

Response: We used a method developed and validated by Presson et al using data from CPRD Aurum and manual case note review. This is the same data source that we use in this study. Their PPV of probable and possible ONJ cases was 92%. Please see Figure 1 in reference (Osteoporos Int. 2025;36(1):47-60) for details. We have added a sentence in methods:

ONJ was ascertained using a two-step algorithm adapted from Persson *et al* with a positive predictive value of 92%.

Unfortunately, data on tooth extraction are not available in the CPRD. This is added a limitation.

Eighth, data on tooth extraction was not available and the association between tooth extraction and ONJ in patients prescribed bisphosphonates ought to be further explored.

4. ONJ precision & rare event methods. ONJ events are extremely few within 2years (0.07/1,000 py after 3y; none after 5y). Use penalized likelihood (Firth) and present wider windows (5–10y) in main text (currently in tables), plus exact counts where disclosure rules allow.
Response: Thank you. The rates of ONJ over longer follow-up were included in the manuscript in results. As we have not undertaken any association analyses for this outcome, we have not undertaken penalized likelihood (Firth).

5. Integrate benefit–harm framing. Place absolute fracture risks (no increase with interruption) alongside absolute ONJ/AFF rates (e.g., AFF 1.93 and 2.50/1,000py; ONJ ≈0–0.07/1,000py over 0–2y) with numbers needed to treat/harm for “continue vs pause.”
Response: Thank you. We previously gave benefit risk framing as advised by the reviewer in our conclusion statement. As there were few ONJ and AFF, we did not examine the association between continue vs. pause bisphosphonates and ONJ or AFF due to lack of statistical power. As a result, we are unable to calculate the NNH.

6. a)Agent specific heterogeneity. You note more ONJ on alendronate and higher AFF on risedronate in some strata;

Response: Yes, there were more ONJ on alendronate and higher AFF on risedronate.

6.b) provide fully adjusted agent specific effects for “continue vs pause,” and interaction P values.
Response: Thank you. We undertook interaction analyses as suggested by the reviewer. There was no evidence of statistically significant interaction.

Please see results:

Interaction tests for age, sex, deprivation, ethnicity, drug type, prior fragility fractures, and prior hip fractures were not significant with P-value 0.788, 0.942, 0.827, 0.301, 0.852, 0.484, 0.167 respectively in the cohort prescribed bisphosphonates for three years and with p-value 0.216, 0.861, 0.896, 0.631, 0.559, 0.853 and 0.632 respectively, in the cohort prescribed bisphosphonates for five years.

7. a) Confounding by unmeasured bone status. Without BMD or vertebral morphometry, risk stratification is limited. Consider proxies (prior vertebral fracture codes, height loss, high dose steroids),
Response: We already considered prior fracture as a stratification factor and corticosteroid prescription as covariates. The reviewer makes an excellent suggestion about assessing height loss as a covariate. We have explored this previously in another study and height is often recorded only once or twice for vast majority of patients, often many years apart. This is typically done when someone joins a GP practice for the first time, and mostly not repeated. We were unable to include height loss as a covariate. We don’t feel residual confounding explains our results given the findings of negative control exposure analysis.

7.b) perform E value.

Response: E value has now been checked. We have added this in methods and in results:

E-value was calculated to assess how strong unmeasured confounding would have to be to negate the observed results on the primary exposure.

The E-value was 1.51 and 1.16 for intermittent prescription and prescription interruption respectively in the three-year cohort; and 1.56 and 1.39 for intermittent prescription and prescription interruption respectively in the five-year cohort.

7.c) perform a negative control outcome analysis (e.g., non skeletal events).

Response: Thank you for this suggestion. It was not possible to undertake a negative control outcome analysis due to constraints of time and funding. A negative control outcome analysis would require us to re-ascertain a new nested case control study population, ascertain all exposures and undertake re-analyses. This would take six months at a minimum and would require additional

funding. We have nevertheless undertaken further analysis to explore the association between negative and positive control exposure and the outcome of interest. This is an acceptable approach and as valid as negative control outcome analysis (doi: 10.1007/s40471-020-00243-4). As mentioned above, there was no association with negative control exposure and the outcome of interest.

8. Outcome definition hierarchy. Report results when restricting fragility fractures to hip only (your sensitivity suggests similar null for interruption; intermittent shows borderline higher hip risk) and make this a co primary or key secondary analysis.
Response: Apologies, a full table of these results is now included. Please see Table S5.

9. Competing risks & mortality. In this elderly cohort, use cause specific or subdistribution hazard models for (atypical femoral) fracture and ONJ across 5–10y horizons to account for death as a competing event.

Response: Thank you for raising this point. As we did not set out to assess the association between any exposure and ONJ, and AFF, the use of cause specific or sub distribution hazard models to account for death as a competing event was not needed. We report incidence rates of these outcomes in which follow-up was censored for loss to follow-up including death. These were purely descriptive analyses.

10. Transparency of code lists. Move the long code lists (TableS1) into a public repository with versioning and upload the analytic code used for ONJ adjudication.
Response: ONJ codes were ascertained from a published code list and are available from Presson et al. The codes in Table S1 have been added to Github (https://doi.org/10.5281/zenodo.18770532) and will also be uploaded to online repository once we are granted access to the website. This has been indicated in the manuscript's data availability section.

Reviewer: 3
Miss Xiaowen Zhang, University of Hong Kong Faculty of Medicine
Comments to the Author
Major:

1. The reliance on MPR ≥67% is unconventional in osteoporosis pharmacoepidemiology. MPR could be inflated in some cases.

Response: We undertook several sensitivity analyses of different levels of MPR. Please also see response to reviewer 1. We have added another limitation:

Additionally, we used MPR to assess exposure status and this could be an overestimate of actual drug consumption. However, any such bias is likely to be non-differential.

2. The 2-year averaging MPR approach obscures some true discontinuation (e.g., a 95-day gap could be missed using MPR). Using the more standard Proportion of Days Covered (PDC) and defining discontinuation via a persistence rule with a pre-specified grace period might be more accurate.

Response: Thank you, we have added this as a discussion point.

The exposure was averaged over two-years (one-year in a sensitivity analysis) which obscured temporary discontinuations. We do not think the duration or timing of such breaks will have a substantial effect on fracture risk as those prescribed bisphosphonates for 3-5 years continue to have suppressed bone turnover markers for 1-2 years after the treatment is discontinued.

3. Handling switches to other anti-osteoporosis medications (e.g., denosumab, teriparatide) was not specified in the methods.

Response: Data on switching to other anti-osteoporosis medications e.g., denosumab, teriparatide was not available as these are almost always hospital only prescribed drugs. Denosumab is very occasionally prescribed from primary care. CPRD only contains details of primary-care prescribed drugs. This was already included as a study limitation. However, we do not think this affects the validity of our findings, as in a cohort with good adherence to oral bisphosphonates for at-least three years, such treatment changes would typically occur after there was a fragility fracture, an outcome at which the follow-up would cease.

4. The nested case-control design offers a powerful opportunity to investigate a dose-response relationship based on the duration of the treatment gap and the timing of the gap, but they were not done in the current study. Exploring these would add novelty to the findings.

Response: As bisphosphonates have strong binding to the bones and exert pharmacologic effects for 1-2 years after discontinuation, we do not think the duration or timing of such breaks in treatment will have any additional effect on fracture risk. Please also see responses to earlier reviewer comments.

5. Important covariates such as BMD, prior fracture severity, physical activity, fall-related risk factors e.g. use of drugs (PPIs, antipsychotics) were not adjusted in the analyses. Although some may be restricted by the data availability (BMD was discussed in the study limitation), it may restrain the interpretation of the results and clinical impact.

Response: Thank you for raising these important issues. We have added these as limitations.

Ninth, we did not have data on prior fracture severity, physical activity, and most risk factors for falls, due to these data not being available in the CPRD. We could not adjust for them in our analyses, and this is a key study limitation. However, there was no evidence of residual confounding on negative control analysis.

6. Outcomes: the authors did hip fracture individually. How about other sites e.g. vertebral fractures?

Response: Thank you. As suggested by the reviewer we have included vertebral fracture as another outcome (Please see Table S6).

Fourth, we considered only (a) hip fracture, and (b) vertebral fracture to define the case status.

There was no association between bisphosphonate interruption and increased risk of vertebral fractures (Table S6).

7. A subgroup analysis by sex would be highly valuable, given the predominant use of bisphosphonates in women.

Response: Thank you. As suggested by the reviewer we have included a subgroup analysis by sex.

Results were similar across both sexes (Table S1).

8. Broaderline OR of hip fracture risk (1.47, 1.00–2.16) and warrants more detailed discussion, and suggest do a female subgroup analysis.

Response: We have added the below discussion point.

There was a borderline increase in the odds of hip fractures with intermittent prescription in the three-year cohort. There was no such association with treatment pause. This could have been a chance finding in a sensitivity analysis and it needs confirmation in future studies.

As suggested by the reviewer we have included a subgroup analysis for women.

There was no association between bisphosphonate interruption and increased risk of hip fracture in women (Table S5).

9. The statement implying “no increase in fragility fractures within two years after discontinuation” may be somewhat strong given the exposure definition, residual confoundings, and borderline hip fracture result. It may be interpreted as a two-year drug gap but the study used MPR.

Response: We agree, and have changed this statement and added another discussion point:

In this nationally representative cohort of people prescribed bisphosphonates for three- and five-years with ≥67% MPR in each year, there was no evidence of increase in the odds of fragility fractures within the next two-years when the bisphosphonate prescription was discontinued, or there was infrequent bisphosphonate prescription. The findings were robust to exposure misclassification and there was no evidence for residual confounding. Nevertheless, these findings should be interpreted with caution as this was an observational study.

Minor comments:

1. Correct typos (e.g., Table 2 title) and ensure the abstract’s ORs for 3-year and 5-year analyses match the main tables.

Response: This has been corrected.

2. In Figure 2, the exclusion criteria list "denosumab," which is not a bisphosphonate.

Response: Apologies, bisphosphonates has been changed to anti-osteoporosis drug.

3. Providing a statistical comparison of ONJ and AFF risk between the discontinuation and continuation groups would be more interesting.

Response: Thank you for this comment. There were too few ONJ and AFF cases to undertake these analyses. However, this has been addressed previously and could be explored in a future study. We have added this as a research recommendation.

A pause in bisphosphonate treatment has been associated with reduced risk of AFF (4), future research should further explore the effect of bisphosphonate discontinuation on risk of ONJ.

Reviewer: 4

Dr. Rebecca Persson, Boston Collaborat Drug Surveillance Program

Comments to the Author

This study in the CPRD assessed the effect of bisphosphonate treatment holidays on fracture risk as well as AFF and ONJ using case-control and cohort analyses. These analyses appear to be well conducted, and this study will add useful information for patients and clinicians. I do have a few comments for the authors' consideration.

Response: Thank you for the positive comments.

Major comments:

1. In the limitations section lines 38-50 the authors write about the possibility of misclassification of exposure due to specialist treatments (which are not captured in the CPRD), in particular, the potential of zoledronic acid or denosumab exposed patients to be misclassified as treatment interrupted patients. This is a concern that requires more consideration than the authors have provided as this misclassification, if extreme, could entirely explain the null observed effect for fracture risk. The authors should consider quantitative bias analysis or other method to assess whether or not their observed results could be a result of exposure misclassification. For example: How many patients from the treatment interruption group would have to have been misclassified for a true effect to be erased? Is there any reason to think misclassification could be different for cases and controls? etc.

Response: Thank you for raising this important point. We have added the below to methods:

We undertook quantitative bias analysis for misclassification of primary exposure.

And results:

Quantitative bias analysis: In a cohort of prescribed bisphosphonates with high levels of MPR for three- (or five-) years, we do not anticipate a switch to second line osteoporosis medications without an intercurrent event i.e., fragility fracture. Similarly, we do not anticipate many patients will experience side-effects of bisphosphonates during established therapy and change treatment. For this reason, we anticipate most patients in the three (or five) years treated cohorts will not be affected by exposure misclassification, and there is no reason to suspect that this will be differential between cases and controls. Nevertheless, assuming a minimum 90% sensitivity and 90% specificity, trapezoidal distribution, the univariate OR (95% CI) accounting for such levels of misclassification would be 1.22 (0.97-1.66) in the three-year cohort and 0.94 (0.73-1.18) in the five-year cohort for interruption versus continued treatment.

2. The authors use the term adherence throughout the paper. It is important for the authors to state at some point in the manuscript that CPRD contains records of prescriptions written, not prescriptions dispensed, and not pills taken and thus that adherence levels have been assumed based on repeated prescriptions.

Response: Thank you. The language has been corrected throughout.

3. Discussion section: “Almost all the AFF and ONJ outcomes were ascertained in hospitalization and mortality records. The positive predictive value of AFF recorded in hospital discharge summaries is reported to range between 90%-92% (27, 28). This increases the validity of our findings.” This is somewhat misleading as references 27 and 28 are validation studies of codes for “Subtrochanteric femur or femoral shaft fracture” and “Subtrochanteric or Diaphyseal Femoral Fractures”, respectively, not AFF specifically. The authors should clarify that all of these cases are assumed to be atypical due to bisphosphonate exposure. It would strengthen their AFF case definition if they excluded any cases with major accidents occurring around the time of the fracture event. However, it is reassuring that the inclusion of any typical femoral fractures as AFF would have overestimated the rates of AFF and thus would not change the overall conclusion that AFF was an uncommon adverse event.

Response: Thank you. There was only 1 such outcome. We have changed the sentence “The positive predictive value of AFF recorded in hospital discharge summaries is reported to range between 90%-92% (27, 28).”

to:

The positive predictive value of subtrochanteric femur or femoral shaft fracture and subtrochanteric or diaphyseal femoral fractures recorded in hospital discharge summaries is reported to range between 90%-92% (27, 28).

We have also added:

There were <5 road traffic accident hospitalisations within 90 days before AFF (exact number suppressed due to CPRD disclosure rules to prevent inadvertent identification of patients with rare outcomes where there are <5 events), and we believe that almost all AFFs could exclusively be attributed to bisphosphonate exposure.

For the reviewers' information we can confirm that there was only one such AFF that was accompanied by hospital admission for a road traffic accident within 90 days (this was on the same day).

4. Results for Incidence of AFF, lines 40-45: the statement that the incidence of AFF as higher with prescription steroids is not consistent with the data presented in Table 3. Please revise.

Response: Apologies, this has been corrected.

Minor comments:

1. Introduction: 2nd sentence is very long and hard to parse. Consider revision.
Response: Thank you. This has been divided into a few sentences.

Bisphosphonates have been used in the management of osteoporosis for over thirty years. They exert a persistent anti-resorptive effect when treatment is discontinued after an initial treatment period of 3-5 years (1-3). Their long-term use has been the associated with atypical femoral fracture (AFF) and osteonecrosis of jaw (ONJ) (4, 5). In order to maximise patient benefit and minimise harm, treatment interruption is recommended. The Food and Drugs Administration, American Society for Bone and Mineral Research, and National Osteoporosis Guideline Group (NOGG) recommend bisphosphonate treatment for an initial period of 3-5 years in those at low-risk, with prolonged treatment for 10-years recommended in those at high risk of fragility fracture (6-9).

2. It is unclear if the >5 year cohort includes patients in the >3 year cohort. Please describe any overlap.

Response: Patients in the 5-year treated cohort were a subset of the 3-year treated cohort. This reflects what would happen in the real world. We have added the below to discussion:

In this study, the 5-year treatment cohort is a subset of the 3-year treatment cohort. Consequently, the results of the 5-year treatment cohort are not independent of the 3-year treatment cohort. However, this reflects real world clinical practice in which patients that have been treated for 5-years, would have also been treated for 3-years.

3. Figure 1: Please clarify index date and exposure assessment period

Response: Thank you. Apart from correcting this figure, the section on case control and exposure window has been rewritten to provide clarity

4. Table 2: Right column header should be "Prior 5-years", correct?

Response: Thank you. This has been amended.

5. Table 3: Clarify with words or improved formatting which analyses are for the 2-year ascertainment period and which are for other ascertainment periods.

Response: Thank you. The 1-year analyses were for sensitivity analysis and they have been included in that section.

Reviewer: 5

Prof. Rafael Perera, University of Oxford

Comments to the Author bmjmed-2025-002085: Fragility fractures, atypical femoral fractures, and osteonecrosis of jaw after initial bisphosphonate treatment for three and five years: nested case-control and cohort studies using linked English primary-care, hospitalisation and mortality data.

Stats Review: The current manuscript presents results from a cohort study based on CPRD (UK primary care) data and two nested case-control studies (varying treatment duration) to estimate the prevalence of fragility fractures (and similar outcomes) and evaluate the impact that stopping (or reducing) bisphosphonate treatment has on the risk of sustaining these types of fractures. The authors find that in this population, the risk of these outcomes do not increase or decrease after treatment discontinuation (or reduction). The findings appear mostly in line with previous research and appear credible. There are some clarifications required in the form the studies are reported which should help put the clinical implications in the right perspective.

Response: Thank you for the positive comments.

Issues:

1. The definition of the cohort is based around a medication possession ratio (MPR) > 2/3 of the time (67%). This measure is a proxy for adherence and is treated as a categorical variable (with some extra sensitivity analyses that modify the threshold at which these categories are defined). The use of categories is likely to reduce the power of the measure to detect different risk levels and there needs to be a justification for this approach. At the same time, perhaps surprisingly, the 'middle category' appears to present an increased risk (but not at the extremes). Some discussion about the reason for this observed difference is required. Regarding this point, please comment about the potential effect that might exist and would not be detectable given the data available (as the confidence intervals for the Odds Ratios are not very precise.

Response: We carefully looked at the tables and did not find any evidence that the middle category patients were at increased risk. We agree that the 95% CI are broad and therefore smaller levels of increased risk from treatment interruption would not have been detected. This is added as a study limitation.

Finally, the 95% CI are broad and therefore smaller levels of increased fragility fracture risk from treatment interruption would not have been detected.

2.- From the description, continuous MPR > 67% appears to be required to enter the nested case-control studies (3 and 5-years). Please clarify what approach is used during the follow up (2 year) period. In particular if different MPR category levels are registered during this period, how was this incorporated into the analysis?

Response: Apologies this was unclear. Patients could have any level of MPR in the follow up (2 year) period. This was used to define the exposure. Please see the sections on exposure and statistical analysis for details. Also, Figure 1 has been redrawn to make this more apparent.

3. - The two nested case-control studies as defined appear to suggest that the 5-year treatment cohort is a subset of the 3-year one. Please clarify if this is correct and comment on what implications this has in relation to the lack of independence of the data and how this should be interpreted.

Response: Thank you. The below sentences have been added to the discussion.

In this study, the five-year cohort is a subset of the three-year cohort. This reflects real world clinical practice in which patients that have been treated for five-years, would have also been treated for three-years. However, because of this, the findings of the two nested case control studies are not completely independent.

4. - Please also provide a justification for the use of a 2-year follow-up.

Response: Thank you, we have added the below sentence to the manuscript.

We used a two-year follow-up as a two-year interruption in treatment is associated with decline in BMD and clinically apparent vertebral fracture, declines after current guidelines recommend an up to two-year pause in bisphosphonate treatment after an initial course of treatment for at-least three years (1, 10, 11).

5. - The fragility fracture outcome appears to potentially occur at any point during the treatment (cohort definition) or follow-up intervals. Please explain if this is the case and also what implications this has for the analysis? Previous fragility fracture does not appear to be one of the covariates included in the model but it would be expected that this would be an important risk modifier for future fragility fractures. Please clarify and justify the approach taken.

Response: The fragility fracture used to define the case control status could only occur during the follow up period i.e., after 3- (or 5-) years of prescription.

Prior fragility fracture (i.e., events during the first 3- (or 5-) years of bisphosphonate prescription) was not considered as a covariate as we thought it would not be a confounder in a population with high levels of MPR during this period, and that adjusting for it could result in overadjustment. We explored this as an interaction term, with a subgroup analysis planned if there was statistically significant interaction observed. We did not find evidence of any such interaction.

Given the reviewer comments, we have included this as a covariate in a sensitivity analysis and the results were unchanged (Please see below). We are happy to add this to the manuscript if the reviewer so advises.

Results from a fully adjusted model that included prior fracture in the cohort prescribed bisphosphonates for three-years: Intermittent prescription aOR 1.15; 95 %CI 0.94, 1.41, Prescription interruption: aOR 1.03; 95 %CI 0.85, 1.25

and five years: Intermittent prescription aOR 0.84; 95 %CI 0.64,1.09, Prescription interruption: aOR 0.88 95 %CI 0.71,1.08.

6. - The statement 'Our findings suggest that in those with prior fragility fractures, treatment interruption for up to two years could be considered after a minimum of three years of treatment.', seems to me to be an overinterpretation of the data. Please provide justification.

Response: We can see your point and have deleted this sentence as it was based on lack of statistically significant interaction between exposure and prior fragility fracture. Thank you for the suggestion. We have added the below discussion point:

The current guidance is for these patients to receive anti-resorptive drug treatment for 10 years and treatment interruption is not specifically recommended to be considered (9). Our findings raise the possibility that such patients could pause treatment for up to two years, perhaps after five years' prescription provided there was good adherence. However, this should be confirmed in another study before changing clinical practice.

7. - For Table S5, the percentage given for BMI category 25-29.9 '(41%)' is incorrect. Please check data in table.

Response: Apologies, this has now been corrected.
